# Mechanistic Basis for In Vivo Therapeutic Efficacy of CK2 Inhibitor CX-4945 in Acute Myeloid Leukemia

**DOI:** 10.3390/cancers13051127

**Published:** 2021-03-05

**Authors:** Morgann Klink, Mohammad Atiqur Rahman, Chunhua Song, Pavan Kumar Dhanyamraju, Melanie Ehudin, Yali Ding, Sadie Steffens, Preeti Bhadauria, Soumya Iyer, Cesar Aliaga, Dhimant Desai, Suming Huang, David Claxton, Arati Sharma, Chandrika Gowda

**Affiliations:** 1 Department of Pediatrics, Pennsylvania State University College of Medicine, Hershey, PA 17033, USA; mreed7@pennstatehealth.psu.edu (M.K.); mrahman@pennstatehealth.psu.edu (M.A.R.); chunhua.song@osumc.edu (C.S.); pdhanyamraju@pennstatehealth.psu.edu (P.K.D.); mehudin@pennstatehealth.psu.edu (M.E.); yding@pennstatehealth.psu.edu (Y.D.); sadiesteffens@eurofinsus.com (S.S.); preeti.bhadauria66@gmail.com (P.B.); iyers@uchicago.edu (S.I.); shuang4@pennstatehealth.psu.edu (S.H.); 2 Department of Medicine, Pennsylvania State University College of Medicine, Hershey, PA 17033, USA; caliaga@pennstatehealth.psu.edu (C.A.); dclaxton@pennstatehealth.psu.edu (D.C.); 3 Department of Pharmacology, Pennsylvania State University College of Medicine, Hershey, PA 17033, USA; ddesai@pennstatehealth.psu.edu (D.D.); asharma@pennstatehealth.psu.edu (A.S.); 4 Department of Medicine, Ohio State University College of Medicine, Columbus, OH 43210, USA; 5 Department of Radiation Oncology, University of Chicago,Chicago, IL 60607, USA

**Keywords:** protein kinase CK2, acute myeloid leukemia, ikaros, bcl-xl, anti-apoptotic gene, CX-4945, transcriptional regulation, patient-derived xenograft, preclinical model, daunorubicin

## Abstract

**Simple Summary:**

Acute Myeloid Leukemia is an aggressive disease with poor outcomes. New targeted therapies that can boost the effects of currently used chemotherapy medications without added toxicity are needed. Targeting an overactive kinase, called the protein Kinase CK2 in AML, helps leukemia cells undergo cell death and helps certain chemotherapy drugs work better. Here, we present evidence that CX-4945, a CK2 inhibitor drug, effectively kills leukemia cells in mouse models and shows the mechanism of action responsible for these effects. Leukemia cells are more sensitive to a decrease in CK2 kinase levels than normal cells. Our results show that inhibiting CK2 kinase makes AML cells more susceptible to anthracycline-induced cell death. Anthracyclines like daunorubicin and doxorubicin are widely used to treat leukemia in children and adults. A rational combination of protein kinase CK2 inhibitors with the standard of care chemotherapy may help treat AML more effectively.

**Abstract:**

Protein Kinase CK2 (Casein Kinase 2 or CK2) is a constitutively active serine-threonine kinase overactive in human malignancies. Increased expression and activity of CK2 in Acute Myeloid Leukemia (AML) is associated with a poor outcome. CK2 promotes AML cell survival by impinging on multiple oncogenic signaling pathways. The selective small-molecule CK2 inhibitor CX-4945 has shown in vitro cytotoxicity in AML. Here, we report that CX-4945 has a strong in vivo therapeutic effect in preclinical models of AML. The analysis of genome-wide DNA-binding and gene expression in CX-4945 treated AML cells shows that one mechanism, by which CK2 inhibition exerts a therapeutic effect in AML, involves the revival of IKAROS tumor suppressor function. CK2 phosphorylates IKAROS and disrupts IKAROS’ transcriptional activity by impairing DNA-binding and association with chromatin modifiers. Here, we demonstrate that CK2 inhibition decreases IKAROS phosphorylation and restores IKAROS binding to DNA. Further functional experiments show that IKAROS negatively regulates the transcription of anti-apoptotic genes, including BCL-XL (B cell Lymphoma like–2 like 1, BCL2L1). CX-4945 restitutes the IKAROS-mediated repression of BCL-XL in vivo and sensitizes AML cells to apoptosis. Using CX-4945, alongside the cytotoxic chemotherapeutic drug daunorubicin, augments BCL-XL suppression and AML cell apoptosis. Overall, these results establish the in vivo therapeutic efficacy of CX-4945 in AML preclinical models and determine the role of CK2 and IKAROS in regulating apoptosis in AML. Furthermore, our study provides functional and mechanistic bases for the addition of CK2 inhibitors to AML therapy.

## 1. Introduction

Acute Myeloid Leukemia (AML) is a heterogeneous hematological malignancy with poor prognosis, despite aggressive therapy, in children and adults alike [1]. Cytotoxic chemotherapy and hematopoietic stem cell transplant remain the mainstay of treatment for AML [2]. Anthracyclines (daunorubicin and mitoxantrone) and cytarabine (Ara-C) form the backbone of AML induction therapy. Although therapies targeting recurrent genetic mutations are being developed, strategies to target non-mutation based vulnerabilities of leukemia cells are mostly unexplored in AML [3]. The therapeutic benefits of selectively targeting constitutively overactive kinases in leukemia, even in the absence of associated genetic mutation, are well established [4].

Protein Kinase CK2, formerly known as Casein Kinase 2 (CK2), is a pleiotropic, constitutively active, serine-threonine kinase essential for cell survival and development [5]. CK2 exists as a tetramer with two catalytic subunits (α or α’) and two regulatory subunits (β). There are two isoforms, CK2α and CK2α’, which are encoded by different genes and have a similar protein structure (except for C-terminus of α subunit) and catalytic activity [6]. CK2 hyperactivity is implicated in the pathogenesis of several cancers, including hematological malignancies such as AML, Acute Lymphoblastic Leukemia (ALL), Chronic Lymphocytic Leukemia (CLL), Chronic Myelogenous Leukemia (CML), and Myeloproliferative Neoplasm (MPN) [7,8,9,10,11]. CK2 represents a key anticancer target that enables leukemia cell survival and proliferation and renders tumor cells highly dependent on its activity [12,13]. CK2 is known to regulate PI3K/PTEN-AKT[14,15], NF-kB (Nuclear Factor-Kappa B) [16], Wnt-B-catenin, and Hedgehog (Hh) [9,14] signaling pathways in AML. The CK2-driven post-translational modification of transcription factors and tumor suppressors such as PTEN (Phosphatase And Tensin Homolog), P53, IKAROS, and PML (ProMyelocytic Leukemia protein) impair their transcriptional activity and promote leukemogenesis [17,18,19,20]. Among the normal karyotype AML cases, those with high CK2 expression were associated with decreased disease-free and overall survival, suggesting that the overexpression of CK2 is a negative prognostic marker in AML [14,16]. Inhibition of CK2, either by siRNAs or the specific inhibitor CX-4945, shows a strong cytotoxic activity in AML [14,15,21]. CX-4945 (Silmitasertib), known by the chemical name 5-(3-Chlorophenyl) amino- benzo[c] naphthyridine-8-carboxylic acid, is a selective, ATP competitive small molecule and an irreversible inhibitor of CK2 with activity against all isoforms [22,23,24].

Although previous studies suggested several pathways through which CK2 inhibitors might operate, the in vivo therapeutic action mechanism for CK2 inhibition in AML is not well understood [11,14,21]. In B-cell acute lymphoblastic leukemia (B-ALL), CX-4945 exerts a therapeutic effect via restoration of the tumor suppressor activity of the IKAROS protein [25]. IKAROS is a DNA-binding protein that regulates its target genes’ transcription via chromatin remodeling [26]. Recent studies demonstrated that the IKAROS tumor suppressor function involves regulating the global epigenomic landscape and chromatin accessibility in acute lymphoblastic leukemia (ALL) [26,27]. Hyperphosphorylation of IKAROS by CK2 impairs IKAROS’ DNA-binding ability and pericentromeric localization, all of which disrupts IKAROS’ functions in transcriptional regulation, cell cycle progression, and T-cell differentiation, and even promotes IKAROS degradation [28,29]. The CK2 inhibition restores IKAROS’ DNA-binding ability and its function as a transcriptional regulator of its target genes [25,30]. IKZF1 recurrent mutations are seen in AML [31]. The impaired IKAROS function is associated with the development of AML, though its potential role in AML tumor suppression is largely unknown [32,33,34,35].

Here, we report that IKAROS is hyperphosphorylated in AML cells with CK2 overexpression. The molecular or pharmacological inhibition of CK2 decreases IKAROS phosphorylation and increases IKAROS binding to target gene promoter regions. Genome-wide DNA-binding and gene expression analyses in CX-4945-treated AML cells revealed a strong IKAROS binding to the BCL-2 family genes. We performed loss of function and gain of function experiments showing that IKAROS represses the transcription of B cell Lymphoma Like–extra-large (BCL-XL) encoded by B cell Lymphoma Like 2–like 1 (*BCL2L1*). Furthermore, we demonstrate that the CK2 overexpression impairs IKAROS’ ability to repress BCL-XL. CX-4945 restores the IKAROS-mediated repression of BCL-XL and sensitizes AML cells to apoptosis. Finally, CX-4945 showed anti-tumor activity and prolonged survival in an AML patient-derived xenograft model. We show that in AML, the central mechanism of BCL-XL repression following the CK2 inhibition is due to the restored IKAROS transcription factor activity. These results provide a mechanistic basis to justify the clinical development of CK2 inhibitors combined with cytotoxic therapy to treat AML.

## 2. Methods and Materials

### 2.1. Cells and Cell Culture

HEK 293T, U937, K562 and THP-1 cells were obtained from the American Type Culture Collection (ATCC) and the German Collection of Microorganisms and Cell Cultures (DSMZ). De-identified patient samples were provided by Loma Linda University (Loma Linda, CA, USA) and collaborators at the Penn State Cancer Institute in compliance with institutional review board regulations. Cells were cultured with RPMI 1640 plus (Mediatech, Manassas, VA, USA) with a 10% heat-inactivated Fetal bovine serum (FBS) (HyClone, Rockford, IL, USA) and 1% penicillin-streptomycin. HEK-293T cells were cultured in DMEM (CellGro) supplemented with 10% FBS. Human AML primary cells (AML-1), previously expanded in mice, were cultured in StemSpan SFEM (Stem Cell Technologies, Cambridge, MA, USA), supplemented with a recombinant human stem cell factor (SCF, 100 ng/mL), IL3 (20 ng/mL), FMS-like tyrosine kinase ligand (FLT3L, 100 ng/mL), G-CSF (20 ng/mL), and GM-CSF (20 ng/mL; Shenandoah Biotechnology, Warwick, PA), as well as 1% penicillin-streptomycin.

### 2.2. Drugs and Reagents

The CK2 inhibitor CX-4945 sodium salt was a gift from Senhwa Biosciences. CX-4945 and daunorubicin hydrochloride were purchased from MedChem Express (Monmouth Junction, NJ, USA).

### 2.3. Human Leukemia Mouse Xenograft Models

U937-GFP-luc cells were transplanted into NRG-S (NOD.Cg-Rag1tm1Mom Il2rgtm1Wjl Tg (CMV-IL3, CSF2, KITLG)1Eav/J) mice via tail vein injection at a dose of 25,000 cells per mouse. The treatment was initiated when a bioluminescence intensity (BLI) signal of 100,000 photons/sec was detected. Mice were randomized based on the average BLI signal and treated with CX-4945 100 mg/kg or a vehicle, given via an oral gavage twice daily for 7 days. Bioluminescence imaging (IVIS 100; analysis with the Living Image software (PerkinElmer)) was used to track the leukemia progression. AML patient samples were selected based on their increased CK2 protein expression (mRNA and protein). Irradiated NRG-S mice were injected with 2 million cells per mouse via the tail vein. Engraftment was defined as 2–5% AML cells (human CD 45+, CD13+, and CD33+, BioLegend, San Diego, CA, USA) in peripheral blood (PB). We started the CX-4945 treatment of 100 mg/kg twice daily after confirming the engraftment, and continued for a total of 21 days. After the treatment period, mice were sacrificed, and mononuclear cells were isolated from the bone marrow, spleen, and peripheral blood. The cell count and flow cytometry analysis were performed (Fortessa; BD, San Jose, CA, USA) using AML antibodies against human CD45+, CD13+, and CD33+ cells. One cohort was monitored for moribund signs, and the survival time was noted.

### 2.4. Apoptosis Assay

Apoptosis assays were performed following the manufacturer’s instructions using the Annexin V-7AAD apoptosis detection kit (Luminex, MCH100105) and analyzed on the Muse Cell Analyzer (Luminex, Chicago, IL, USA). Briefly, 100 μL of cells were added to 100 μL of reagent, incubated for 20 min at room temperature, and analyzed.

### 2.5. Proliferation and Cytotoxicity Assays

The colorimetric WST-1 cell proliferation assay (Roche Diagnostics GmbH, Manheim, Germany) was performed in 96-well white clear-bottom plates (Costar, 3610) in quadruplicate experiments, according to the manufacturer’s instructions. Absorbance at 440 nm (reflects the number of viable cells) was measured using a BioTek Synergy Mx plate reader.

### 2.6. Western Blot

Cells were treated with 5 and 10 µM of CX-4945 or the vehicle control for 48 h and the whole-cell lysate was collected. Briefly, samples were prepared on ice using lysis buffer containing 10 mM Tris-HCl (pH 7.4), 5 mM MgCl_2_, 1% Triton X-100, 100 mM NaCl, 10 mM NaF, 1 mM Na_3_VO_4_, and a protease inhibitor cocktail. The protein was quantified using the Bradford assay and used for Western blot analysis and immunoblotting. The antibodies used for the Western blot assay are listed in Table S1.

### 2.7. In Vitro Phospho-IKAROS Labeling

Cells were treated with different doses (5 or 10 μM) of CX-4945 for 48 h. Cells were washed twice with phosphate-free RPMI 1640 and incubated with 0.5 mCi/ml [32P] orthophosphate (PerkinElmer, Waltham, MA, USA) in a phosphate-free RPMI 1640 medium for 6 h. Cells were collected and washed twice with cold PBS and the nuclear protein was extracted using the NE-PER Nuclear and Cytoplasmic extraction reagent kit (Thermo Fisher Scientific, Waltham, MA, USA). The lysis buffer was supplemented with a protease and phosphatase inhibitor cocktail (Thermo Fisher Scientific). Then, IKAROS was immuno-precipitated using the Dynabead Protein G Immunoprecipitation Kit (Thermo Fisher Scientific) according to the manufacturer’s protocol. Briefly, lysates were incubated with the Dynabeads Protein G and IKAROS antibody (Proteintech, Rosemont, IL, USA) complex for 2 h on a rotator-mixer. IKAROS was eluted, separated by SDS-PAGE, transferred to a PVDF membrane, and imaged by radiography.

### 2.8. Colony Formation Assay

Cells were pretreated with CX-4945 alone or combined with daunorubicin, or the vehicle control for 48 h. Cells were washed with PBS and plated in triplicate in a 6-well plate containing the 1.1 ml MethoCult H4100 medium (STEMCELL Technologies, Vancouver, Canada) supplemented with RPMI-1640 and 10% FBS for U937 cells, as well as a MethoCult H4034 Optimum (STEMCELL Technologies) medium for AML-1 cells. Colonies were propagated for 7–14 days in an incubator at 37 °C and 5% CO_2_. Colonies were counted under an inverted light microscope. Colonies that contained around 50 cells or more were counted for analysis.

### 2.9. Gene Expression Analysis by qRT-PCR

Total RNA (ribonucleic acid) was isolated from cells using the QIAshredder and RNeasy Mini Kit (QIAGEN, Germantown, MD, USA). Complementary DNA (cDNA) was generated from 1 μg of total RNA using the Superscript First-Strand Synthesis System (Invitrogen). The qRT-PCR was performed using a StepOne Plus real-time PCR machine (Applied Biosystems) with PerfeCTa SYBR Green FastMix (Quanta Biosciences). Values were normalized to 18 s RNA and the relative expression values were determined by the 2-ΔΔCt method. 

Primers used for target gene studies include:

BCL2L1-5′-TTGGATGGCCACTTACCTGAAT-3′; BCL2L1-Rev 5′-CCGCCGTTCTCCTGGAT-3′; CK2α-5′-AGCGATGGGAACGCTTTG-3′; CK2α-Rev 5′-AAGGCCTCAGGGCTGACAA-3’; 18s-For 5’-GTAACCCGTTGAACCCCATT-3′ 18s-Rev5′-CCATCCAATCGGTAGTAGCG-3′.

### 2.10. Luciferase Assay

The luciferase assay was performed using the LightSwitch Luciferase Assay System (SwitchGear Genomics). Human embryonic kidney (HEK)-293T cells were seeded into 24-well plates. HEK-293T cells do not have endogenous IKAROS. After 24 h, cells were transiently transfected with 0.15 μg of indicated promoter-reporter constructs or pROM vector and 0.15 μg of pcDNA3.1-IKAROS or pcDNA3.1 vector in triplicate experiments for each group using lipofectamine 2000 (Invitrogen, Carlsbad, CA, USA) according to manufacturer’s instructions. According to the manufacturer’s instructions, 24 h after transfection, cells were lysed in 100 μL of a LightSwitch Assay Solution (SwitchGear Genomics, Carlsbad, CA, USA) and rocked at RT for 30 min. Lysates were measured by a luminometer (Promega GloMax 20/20 Luminometer). Luciferase activities were calculated as a fold change relative to the vector-only cells and normalized to pcDNA3.1 vector readings. All transfection and reporter assays were performed in triplicate experiments.

### 2.11. Quantitative Chromatin Immunoprecipitation

IKAROS qChIP assays were performed as described previously[30]. Primers used for the qChIP experiments are listed in the Table S2. 

### 2.12. Statistical Analysis

Graphed data are represented as mean values with bars representing the standard deviation (mean ± SD) of three technical replicates and at least two independent experiments. Statistical analysis was performed using GraphPad Prism 9.0.0. P-value summaries are as follows: *p* > 0.05 (ns); *p* ≤ 0.05 (*); *p* < 0.01 (**); *p* < 0.001 (***); *p* < 0.0001 (****). Statistical significance in Figure 3B,E was performed using the one-way analysis of variance (ANOVA). Statistical analysis for all other column graphs used multiple two-tailed t-tests by the Holm-Sidak method, with alpha = 0.05. Each row (representing a cell line or drug concentration) was analyzed individually, without assuming a consistent Standard Deviation (SD). The number of t-tests per analysis was dependent on the number of conditions. The qChIP values where the signal was more than 2-fold greater than the background anti-Immunoglobulin G (anti-IgG) level were analyzed. The Kaplan-Meier method and the log-rank test was used to perform the survival analysis and compare survival differences (Figures 2G and 3C).

## 3. Results

### 3.1. High Baseline Expression of CK2, Bcl-xl, and p-IKAROS in Myeloid Leukemia Cells Compared to Normal Hematopoietic Stem Cells

**Figure 1:**
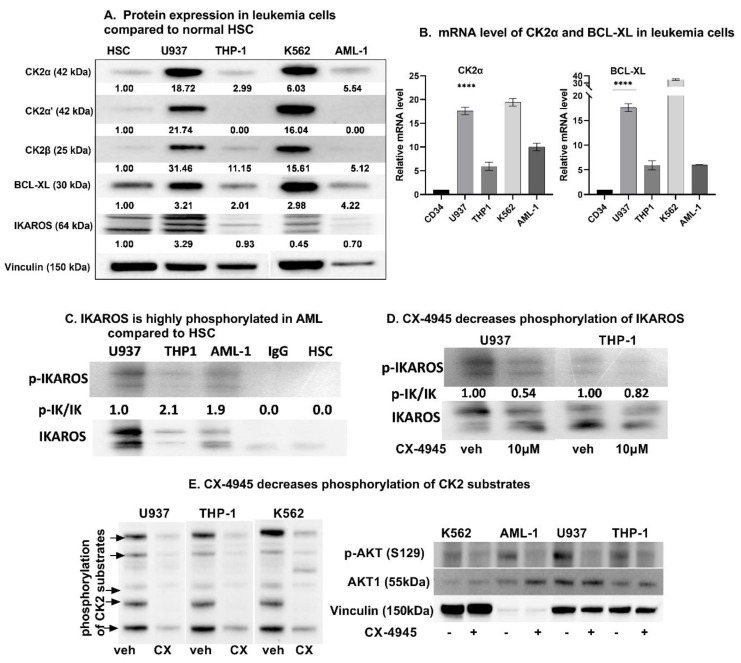
CK2 overexpression in AML cells causes IKAROS phosphorylation which is reversed by CK2 inhibitor, CX-4945. (**A**) Baseline protein levels of CK2α, CK2α’, CK2β, IKAROS and BCL-XL in the myeloid leukemia cell panel [U937, THP-1, K562, and primary AML cells (labelled AML-1) were measured by western blot and compared to CD34+ Hematopoietic Stem Cells (HSC). Multiple IKAROS bands represent Ikaros isoforms 1 and 2. (**B**) qRT-PCR showing mRNA level of CK2α and BCL-XL in various AML cells compared to CD34+ HSC. **** (*p* < 0.0001). (**C**) Radio-blot showing increased Phosphorylated IKAROS (p-IKAROS) in AML cells compared to HSC. (**D**) Radio-immunoblot showing dose-dependent decrease in Phospho-IKAROS level following CX-4945 treatment. U937 and THP-1 were treated with 10 µM and AML-1 was treated with 5μM (IC50–3.791 μM) CX-4945 for 48 h. IC50 values of CX-4945 treated cells are shown in Figure S2. (**E**) Western blot showing decrease in amount of phosphorylated CK2 substrates with molecular masses of approximately 175, 120, 80, 70 and 56 kDa as indicated by arrows (left panel) and western blot showing phosphorylation extent of specific CK2 substrate, AKT1 at Ser129 (right panel).

In line with the previously published data [15,16,36], we observed that CK2 is overexpressed in myeloid leukemia cells and primary cells with various cytogenetic features compared to normal hematopoietic cells (CD34+ HSC). A table with descriptions of each cell type is included in supplemental file (Table S3). We examined protein and mRNA levels of CK2α, CK2α’, CK2β, and BCL-XL in a panel of myeloid leukemia cells, using Western blot (Figure 1A) and qRT-PCR, respectively (Figure 1B). The CK2α and BCL-XL expression were higher in AML cells as compared to CD34+ HSC (Figure 1A and Figure S1). The expression of CK2, BCL-XL, and IKAROS were variable across different AML cells. AML cells showing high BCL-XL expression in the absence of high CK2 expression (as in AML-5 and AML-6 in Figure S1) suggest other possible mechanisms influencing the expression of BCL-XL in AML cells such as cytogenetic alterations, relapse disease, and chemotherapy exposure. CD34+ HSCs were obtained from different sourses such as the umbilical cord blood (UCB) and granulocyte colony stimulating factor (GCSF) treated peripheral blood collection (PB). The CK2 and BCL-XL expression were noted to be significantly and uniformly lower in CD34+ from PB and UCB compared to the leukemia cells. We chose U937, THP1, and AML-1 (primary AML cell) for our experiments due to the high expression of CK2α. Phosphorylated-IKAROS was also measured in the studied cells using a radio-immunoblot. IKAROS was observed to be highly phosphorylated in AML cells compared to HSC (Figure 1C). 

### 3.2. CK2 Inhibition Decreases IKAROS Phosphorylation and CK2 Cellualr Activity

In B-ALL, IKAROS is hyperphosphorylated by CK2 [37,38]. We tested the effect of CK2 inhibition with CX-4945 on IKAROS phosphorylation in AML cells. Compared to the untreated cells, the treatment with CX-4945 for 48 h resulted in a reduction in IKAROS phosphorylation (Figure 1D).

We measured the CK2 activity indirectly by using an antibody against the phospho-CK2 substrate (motif pS/pTDXE) to measure the amount of phosphorylated CK2 substrates [39,40,41,42,43]. The CK2 substrates contain multiple acidic residues (Asp and Glu) located downstream of the phosphorylated serine or threonine residues. The consensus sequence for CK2 substrates is pS/pTD/EXD/E with the most crucial residue at the +3 position followed by the residue at the +1 position. CX-4945 reduced the CK2 activity in a dose-dependent manner 48 h post-treatment as determined by immune-blotting with an antibody that recognizes phosphorylated CK2 sites in multiple proteins (Figure 1E, left panel). AML cell lines showed a reduction in multiple phospho-CK2 substrates with molecular masses of approximately 175, 120, 80, 70, and 56 kDa as indicated by arrows in Figure 1E, left panel. We also performed the Western blot analysis to evaluate the phosphorylation extent of a specific CK2 substrate, AKT1 at Ser129 [44,45]. Results showed a decrease in the p-AKT(S129) level following the CX-4945 treatment (Figure 1E, right panel).

### 3.3. Inhibition of CK2 Represses BCL-XL Expression

**Figure 2:**
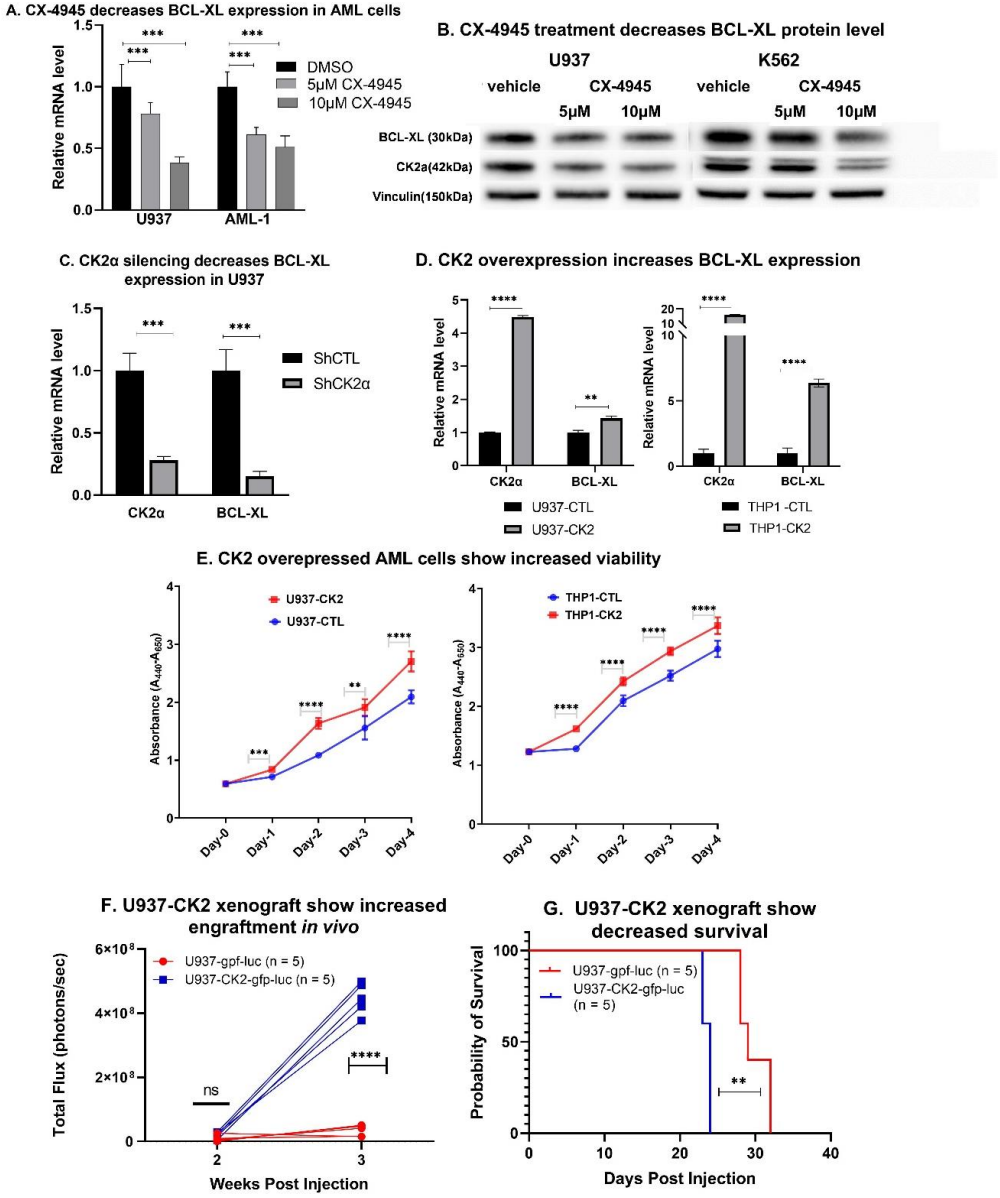
CK2 inhibition suppresses BCL-XL expression in AML cells and xenograft model. (**A**) U937 and AML-1 were treated with 5 and 10 µM concentration of CX-4945 for 48 h before RNA and protein extraction. mRNA level of BCL-XL was measured by qRT-PCR. (**B**) U937 and K562 cells were treated with 5 and 10 µM of CX-4945 for 48 hours and protein was extracted. BCL-XL protein level was measured by western blot. (**C**) Downregulation of CK2α in U937 was achieved using shRNA. Validation of CK2α protein knockdown in U937-shCK2α cell line is shown in Figure S3. qRT-PCR shows mRNA level of CK2α and BCL-XL in CK2α shRNA treated U937 cells. (**D**) Overexpression of CK2α was achieved by retroviral transduction of U937 cells. Validation of CK2α protein overexpression is shown in Figure S6. qRT-PCR showed increased BCL-XL mRNA level in CK2α overexpressed U937 (left panel) and THP-1 cells (right panel). (**E**) WST assay showing increased cell viability in CK2α overexpressing U937 and THP-1 cells. U937 cells transduced with retroviral vector CK2α-gfp-luc were transplanted into NRG-S mice. 25,000 cells were injected via the tail vein. Bioluminescence imaging using IVIS 100 was obtained weekly following transplant. (**F**) Bioluminescence signal in xenograft mice engrafting U937-gfp-luc cell or control (U937-ctl-gfp-luc) shown at week 2 and 3 post transplantation. (**G**) Kaplan Maier plot showing survival probability in U937-CK2-gfp-luc cells vs control. *P*-value summaries are as follows: *p* > 0.05 (ns-not significant); *p* < 0.01 (**); *p* < 0.001 (***); *p* < 0.0001 (****).

We studied the effect of CK2α downregulation and pharmacological inhibition of CK2α on BCL-XL expression. We achieved a pharmacological inhibition of CK2 using a specific inhibitor, CX-4945. The treatment of U937 and AML-1 with CX-4945 showed dose-dependent decreases in BCL-XL mRNA (Figure 2A) and protein levels (Figure 2B). Downregulation of the CK2α was achieved by treating U937 cells with a CK2α specific short hairpin (sh) RNA (Figure S3). CK2α silencing decreased the expression of BCL-XL at mRNA (Figure 2C) and protein levels (Figure S4), as well as decreased IKAROS phosphorylation (Figure S5). Next, we transduced U937 and THP-1 cells with a retrovirus expressing CK2α or the empty vector (as a negative control). Increased CK2α mRNA (Figure 2D) and protein were confirmed (Figure S6). The overexpression of CK2α showed increased BCL-XL mRNA levels in U937 and THP-1 (Figure 2D). We did not observe a significant change in IKAROS phosphorylation or the BCL-XL protein level in CK2 overexpressing U937 cells (Figures S4 and S5). This may be due to the already high level of IKAROS phosphorylation by high baseline CK2 in these cells. Phenotypically, CK2α overexpressing U937 and THP-1 cells showed increased cell viability and proliferation compared to the control cells (Figure 2E). We developed a cell line derived xenograft mouse model by transplanting luciferase labeled U937 cells transduced with CK2α-gfp-luc or the control vector (ctl-gfp-luc) into immunocompromised (NRG-S) mice via tail vein injection. AML (U937-CK2α-gfp-luc) cells overexpressing CK2α showed a robust engraftment as shown by the increased bioluminescence intensity (Figure 2F). Moreover, mice engrafted with CK2α overexpressing AML cells showed decreased survival compared to the control group (Figure 2G).

These results demonstrate that the CK2α downregulation and pharmacological inhibition of CK2 results in decreased IKAROS phosphorylation and BCL-XL repression in AML cells. The overexpression of CK2α promotes cell viability in vitro, and accelerates leukemia progression in vivo likely by increasing the expression of anti-apoptotic BCL-XL. 

### 3.4. CX-4945 Treatment Shows Therapeutic Efficacy in AML Patient-Derived Xenografts

**Figure 3:**
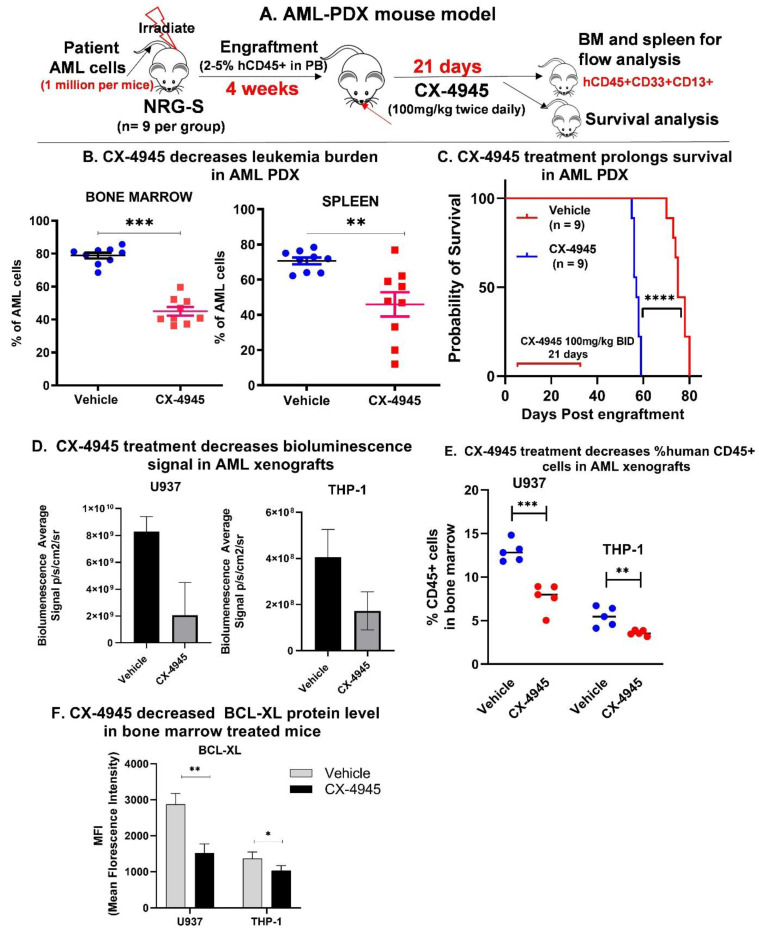
CX-4945 treatment shows therapeutic efficacy in AML Patient Derived Xenograft (PDX). (**A**) Schema showing AML PDX generation and treatment. AML-1 PDX was developed by injecting NRG-S mice with one million cells per mouse via the tail vein. Treatment was started when 2–5% human CD45+ cells were detected in peripheral blood (PB). Mice received 100 mg/kg of CX-4945 via gavage twice daily for 21 days. (**B**) Percent AML (human CD45+, CD13+,CD33+) in bone marrow (BM) and spleen of treated and untreated AML-1 PDX mice by flow cytometry. (**C**) Kaplan Maier plot showing survival probability of treated and untreated AML-1 PDX mice. Cell line derived xenograft (CDX) mouse models was developed using luciferase, and green fluro-protein (gfp) labeled U937 cells (U937-gfp-luc) or THP-1 cells (THP1-gfp-luc) transplanted into immunocompromised NRG-S mice as described in methods. Mice were treated with vehicle or CX-4945 at a dose of 100 mg/kg twice daily via gavage for up to 7 days. (**D**) Engraftment was monitored using bioluminescence imaging shown as the mean of the total flux in photons/second of mice in each group. (**E**) Following treatment, mice were sacrificed, and bone marrow mononuclear cells were collected. Human CD45+ cells in bone marrow were measured using flow cytometry. Flow cytometry using conjugated BCL-XL antibody was used to quantify intracellular BCL-XL protein level in FACS enriched human CD45+cells in the bone marrow of treated and untreated mice. Figure S6 shows histogram of BCL-XL and CK2α protein level in vehicle and CX-4945 treated U937 and THP-1 CDX. (**F**) Mean fluorescence intensity(MFI) is graphed, showing decreased BCL-XL protein level. *P*-value summaries are as follows: *p* > 0.05 (ns-not significant); *p* ≤ 0.05 (*); *p* < 0.01 (**); *p* < 0.001 (***).

We developed a patient-derived xenograft (PDX) model by transplanting previously expanded human primary AML cells (AML-1) into irradiated NRG-S mice via tail vein injection at a dose of 2 million cells per mouse. These mice were treated with CX-4945 orally at 100 mg/kg twice daily for 21 days (Figure 3A). After the treatment period, one cohort of mice was sacrificed to measure the human leukemia burden in the bone marrow and spleen using antibodies against human AML immunophenotypic cell surface markers (hCD45+, CD13+, CD33+). The CX-4945 treated mice showed significantly less leukemia burden (Figure 3B) and prolonged survival (Figure 3C) than the untreated group.

### 3.5. CX-4945 Treatment Decreases BCL-XL Expression In Vivo

We developed a cell line derived xenograft mouse model by transplanting luciferase labeled U937 and THP-1 cells (U937-GFP-luc and THP-1-GFP-luc) into NRG-S mice via tail vein injection. Engraftment was confirmed by bioluminescence imaging. Mice were treated with CX-4945 orally at 100 mg/kg twice daily for 7 days. The CX-4945 treated mice showed decreased BLI (Figure 3D). After the treatment period, mice were sacrificed, and human CD45+ cells were collected from the bone marrow. The CX-4945 treated mice showed decreased total human CD45+ cells compared to the untreated mice (Figure 3E). Intracellular protein quantification of BCL-XL and CK2α in leukemia cells from the bone marrow of treated and untreated mice was done using flow cytometry. AML cells from the mice treated with CX-4945 showed decreased BCL-XL protein levels compared to cells from the untreated mice (Figure 3F and Figure S7) in both U937 and THP-1 xenograft models. We also observed a mild decrease in the CK2α level following the CX-4945 treatement which may suggest that BCL-XL depletion by CX-4945 may be detrmined by the CK2 protein amount (Figure S7). These findings of CK2α protein depletion were not noted in the AML-PDX treated with CX-4945, as shown later. Interestingly, the complete blood count obtained from these mice did not show thrombocytopenia (Table S4). These results confirm that CK2 inhibition by CX-4945 decreases BCL-XL expression in vivo and demonstrates the in vivo anti-leukemia effect of CX-4945 in an AML xenograft model.

### 3.6. CK2 Inhibitor Increases IKAROS DNA-Binding to BCL-XL

**Figure 4:**
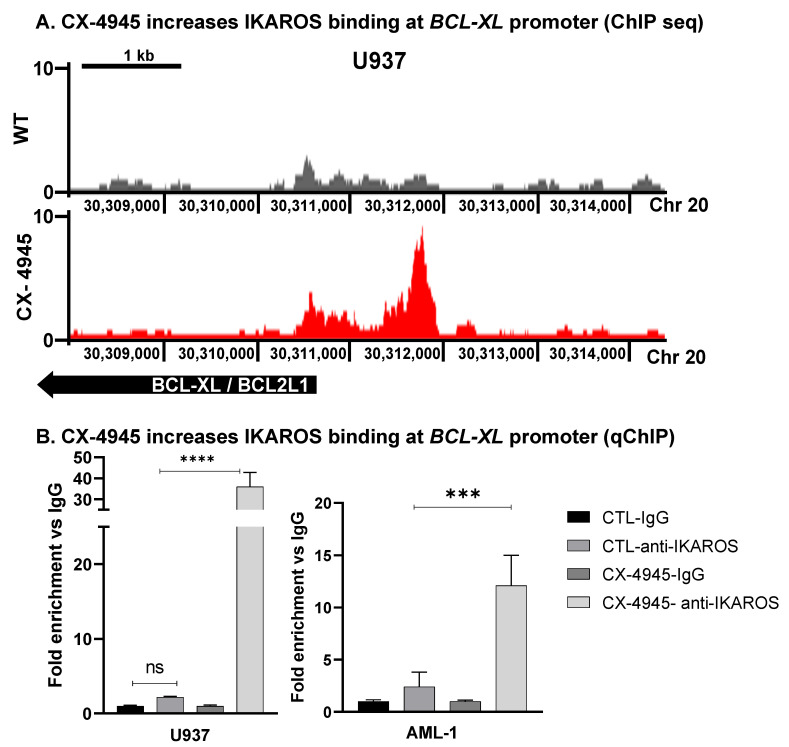
CK2 inhibitor increases IKAROS DNA-binding to *BCL-XL*. Chromatin immunoprecipitation (ChIP) followed by next-generation sequencing (ChIP-seq) and analysis of genome-wide occupancy of IKAROS was performed on U937 following the CX-4945 treatment at 10 µM concentration for 72 h. A change in IKAROS binding to promoter regions of the *BCL-XL* gene was analyzed following the CX-4945 treatment. (**A**) A chIP-seq signal map for IKAROS binding to the *BCL-XL/BCL-*2L1 promoter region in U937-untreated labeled as WT (wild-type) (top panel) and CX-4945 treated U937 (bottom panel). Y-axis represents the log-2-fold change enrichment of IKAROS binding (** *p* < 0.01). (**B**) U937 and primary AML cells (AML-1) cells were treated with 10 and 5 μM of CX-4945, respectively (based on the IC50 value) for 48 and 72 h. IKAROS binding to the *BCL-XL* promoter region was confirmed using the qChIP assay in WT and CX-4945 treated cells. Binding at 72 h was not significantly increased compared to the 48 h treatment (not shown in the graph). Results are the mean +/– SD of three independent experiments. P-value summaries are as follows: *p* > 0.05 (ns-non significant); *p* < 0.001 (***); *p* < 0.0001 (****).

The IKAROS transcription factor binds to upstream regulatory elements of target genes and activates or represses their expression via chromatin remodeling [26,27]. Regulatory functions of the IKAROS transcription factor in AML are not known. In ALL, CK2 is the major kinase that phosphorylates IKAROS [37,38]. In AML, where the *IKZF1* loss of function mutations is rare, hyperphosphorylation by CK2 can impair IKAROS’ DNA-binding ability and subsequent regulatory functions. Using chromatin immunoprecipitation followed by highly parellel DNA sequencing (ChIP-seq), we did a global analysis of changes in the genome-wide DNA-binding of IKAROS in AML cells following the treatment with CX-4945. U937 cells were treated with 10 µM of CX-4945 for 72 h to assess IKAROS mediated epigenomic changes. A link to access the ChIP-seq data in the UCSC browser is provided in the supplemental material. Here, we show that the binding of IKAROS to the promoter region of the *BCL2L1* (*BCL-XL*) gene increases after the CX-4945 treatment (Figure 4A). We confirmed IKAROS binding at the *BCL-XL* promoter region using a quantitative chromatin immunoprecipitation assay (qChIP). The CX-4945 treatment showed increased binding of IKAROS to the *BCL-XL* promoter in U937 (Figure 4B) and primary AML cells following 72 h of treatment (Figure 4B, right panel). 

### 3.7. IKAROS Represses BCL-XL Expression

**Figure 5:**
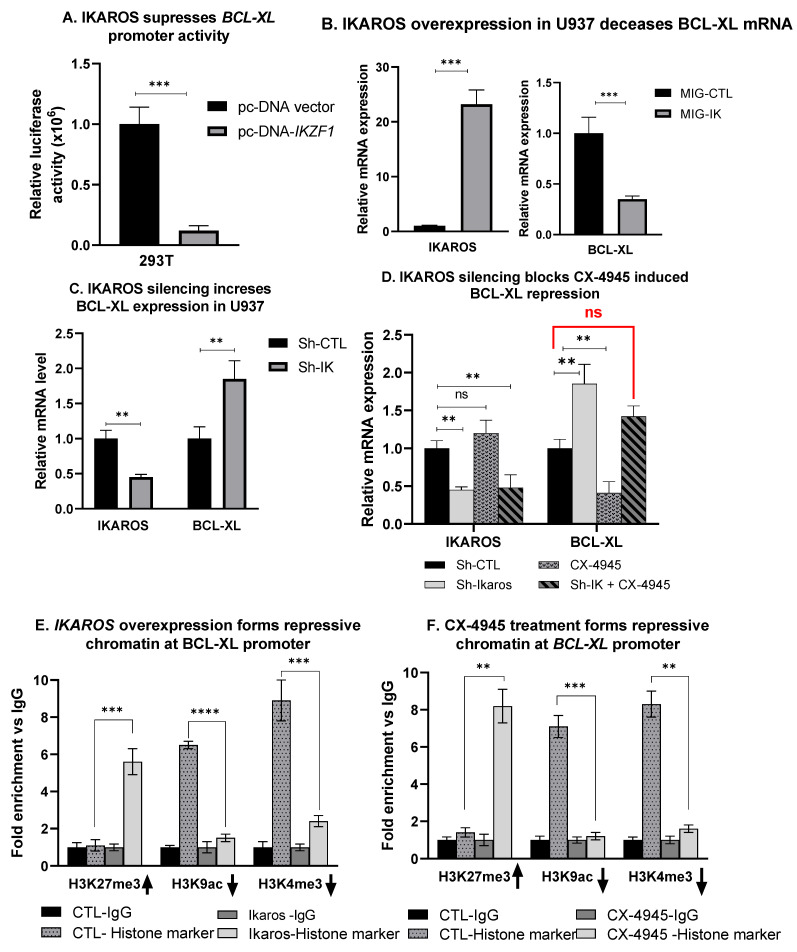
IKAROS represses *BCL-XL* gene transcription in AML. The luciferase reporter assay was performed on HEK-293T cells transfected with *IKZF1* plasmid (pcDNA-IK) or the control vector. Result in (**A**) shows repression of the *BCL-XL* luciferase promoter construct by the IKAROS-expressing vector pcDNA3.1-IK in comparison to the pcDNA3.1 empty vector control in HEK-293T cells. The luciferase activity was normalized to pcDNA3.1 and pROM empty vector controls. (**B**) U937 cells were transduced to express *IKZF1* (MIG-IK) or with an empty vector (MIG-CTL). The relative mRNA expression of IKAROS and (left panel) BCL-XL (right panel) were assessed using qRT-PCR. (**C**) U937 cells were treated with IKZF1 shRNA (shIK) or scramble shRNA control (shCTL). The relative expression of IKZF1 (left panel) and BCL-XL (right panel) assessed by qRT-PCR. (**D**) U937 cells were treated with IKZF1 shRNA (shIK) or scramble shRNA control (shCTL). IKAROS knockdown U937 cells were then treated with 10 µM of CX-4945 for 48 h. Changes in *BCL-XL* gene expression were measured using qPCR. Figure 5E,F shows the qChIP assay showing an enrichment of histone markers at the *BCL-XL* promoter. The qChIP assay was performed using (**E**) IKAROS overexpressing (MIG-IKZF1) U937 cells and (**F**) CX-4945 treated U937 cells (10 µM for 48 h) to determine the fold enrichment of histone markers, H3K27me3, H3K9ac, and H3K4me3 at the *BCL-XL* promoter and compared to the control cells. P-value summaries are as follows: *p* > 0.05 (ns- not significant); *p* < 0.01 (**); *p* < 0.001 (***); *p* < 0.0001 (****). Results are the mean +/– SD of three independent experiments.

We used a luciferase reporter assay to determine if IKAROS binding to the *BCL-XL* promoter region alters *BCL-XL* gene expression. The transient co-transfection of the *BCL-XL* promoter region fused with the reporter gene and *IKZF1* in HEK 293T cells. HEK293T cells do not have endogenous IKAROS making it an ideal system to study the effect of *IKZF1* on our promoters of interest. The human embryonic kidney 293T in human cell line cells stably express the SV40 large T antigen, increasing transfection, and transduction efficiency. The results show that IKAROS represses the *BCL-XL* promoter activity (Figure 5A). Next, we performed the IKAROS loss of function and gain of function experiments to confirm the effect of IKAROS on BCL-XL expression in AML cells. The overexpression of IKAROS in U937 cells was achieved by transduction of the retrovirus expressing wild type *IKZF1* and empty vector as a negative control (Figure 5B, left panel). Increased *IKAROS* mRNA (Figure 5B) and protein (Figure S8) were confirmed. The overexpression of IKAROS in U937 cells resulted in decreased BCL-XL mRNA levels (Figure 5B, right panel) but not the protein level (Figure S8). This could be due to the high level of CK2 present in U937 cells that phosphorylate the overexpressed IKAROS protein. IKAROS silencing was achieved by treating U937 cells with *IKZF1* shRNA (Figure 5C, left panel). The U937 treated with scramble shRNA (sh CTL) was used as a control. IKAROS knockdown resulted in increased mRNA levels of BCL-XL (Figure 5C, right panel). These results establish the role of IKAROS as a transcriptional repressor of BCL-XL in AML.

### 3.8. CX-4945-Induced BCL-XL Repression Is Mediated via IKAROS 

The functional alteration of IKAROS due to phosphorylation by CK2 impairs its regulatory functions as a tumor suppressor in leukemia [38]. CK2 inhibition results in the redistribution of genome-wide DNA-binding of IKAROS to promoter regions of its target genes, restores IKAROS’ regulatory activity, and revives the tumor suppressor function [25]. Since CK2 has multiple substrates, IKAROS being one, we wanted to test whether silencing IKAROS would affect a CX-4945-induced decrease in the BCL-XL expression. We treated U937 cells with *IKZF1* shRNA and CX-4945, and measured BCL-XL mRNA levels. IKAROS silencing blocked the CK2 inhibitor’s ability to induce BCL-XL repression (Figure 5D). This result confirms that the CK2-IKAROS axis is one of the major, if not the only, BCL-XL repression mechanisms by CX-4945. 

### 3.9. IKAROS Represses BCL-XL via the Formation of Repressive Chromatin

Here, we investigated the mechanism by which IKAROS represses its target gene, *BCL-XL,* in AML. IKAROS regulates the transcription of its target genes via chromatin remodeling. Chromatin remodeling often involves chemical modifications of histone protein present in chromatin (i.e., methylation and acetylation). The modification of “histone mark” influences gene expression by changing how accessible the chromatin is to the transcription. The histone mark is a specific modification (acetylation or methylation) of a specific histone protein. Acetylation of lysine 9(K9) of the H3 histone protein is noted as H3K9Ac. Methylation of lysine 27 (K27) of H3 histone is noted as H3K27me3. Enrichment of H3K9 acetylation (H3K9Ac) is an indicator of open and active chromatin. Enrichment of H3K27 tri-methylation (H3K27me3) is a marker of closed and repressive chromatin. Repressive chromatin signature is noted as an enrichment of Histone (H) 3 lysine (K) 27 tri-methylation (H3K27me3) and loss of H3K9 acetylation (H3K9ac) and H3K4 tri-methylation (H3K4me3) [26]. We performed serial qChIP assays on CX-4945 treated U937 cells to evaluate the presence of histone markers indicating a change in the chromatin signature at the *BCL-XL* promoter region. 

Results show that following the treatment with CX-4945, there is an enrichment of the H3K27me3, loss of H3K9ac, and H3K4me3 histone marker (Figure 5F). A similar enrichment pattern was noted at the *BCL-XL* promoter in U937 cells with forced IKAROS overexpression (Figure 5E). IKAROS represses the *BCL-XL* gene’s transcription by inducing the formation of repressive chromatin at the *BCL-XL* promoter.

### 3.10. CK2 Regulate AML Cell Sensitivity towards Daunorubicin

**Figure 6:**
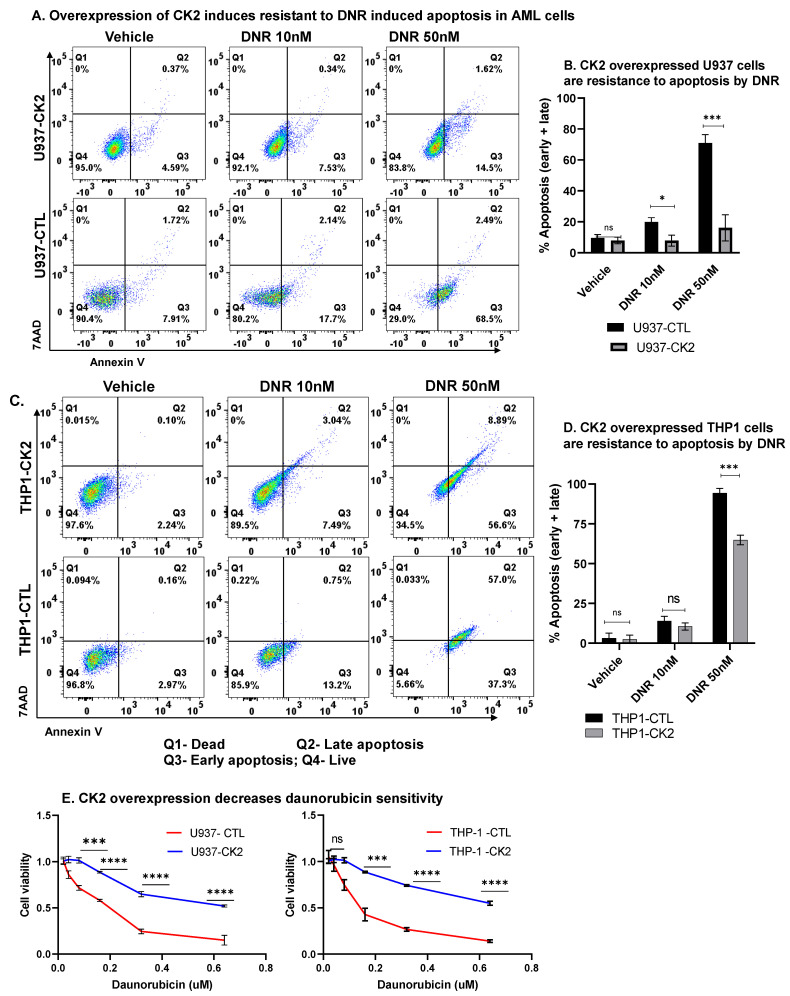
CK2 and IKAROS regulate sensitivity towards daunorubicin. CK2 overexpressing U937 (**A**,**B**) and THP-1 (**C**,**D**) were treated with 10 nM or 50 nM of daunorubicin for 48 hand stained with 7-AAD and Annexin V for flow cytometry to determine apoptosis. Flow plots (**A**,**C**) and percent apoptotic cells (early + late apoptosis) are shown in (**B**,**D**). Flow plots showing representative results from three replicates. The percentage of cells in the right upper and lower quadrant of each flow chart represents the percentage of late and early apoptotic cells, respectively. Q1-dead, Q2-late apoptosis, Q3-early apoptosis, Q4-live. (**E**) Cytotoxicity and drug response measured by MTT assay after treating CK2 overexpressing U937 and THP-1 cells and respective controls with various concentrations of daunorubicin for 48 h. *p* > 0.05 (ns); *p* < 0.05 (*); *p* < 0.001 (***); *p* < 0.0001 (****).

Daunorubicin (DNR) is one of the most commonly used cytotoxic therapies for AML that induces DNA damage and apoptosis. AML cells often overexpress BCL-XL and other anti-apoptotic genes as one mechanism of chemoresistance. As shown in Figure 2A,B, the CK2 inhibition decreases BCL-XL expression. Here, we tested whether the CK2 inhibition affects AML cells’ sensitivity to DNR-induced apoptosis, and, conversely, whether CK2α overexpression in AML cells confers resistance to DNR induced apoptosis. CK2α overexpressing AML cells were resistant to apoptosis (Figure 6A–D) and showed decreased drug response (Figure 6E) following the DNR treatment compared to the control cells. Overall, these results confirm the role of CK2 overexpression in inducing resistance towards cytotoxic therapy in AML.

### 3.11. CK2 Inhibition Augments Daunorubicin Drug Response

**Figure 7:**
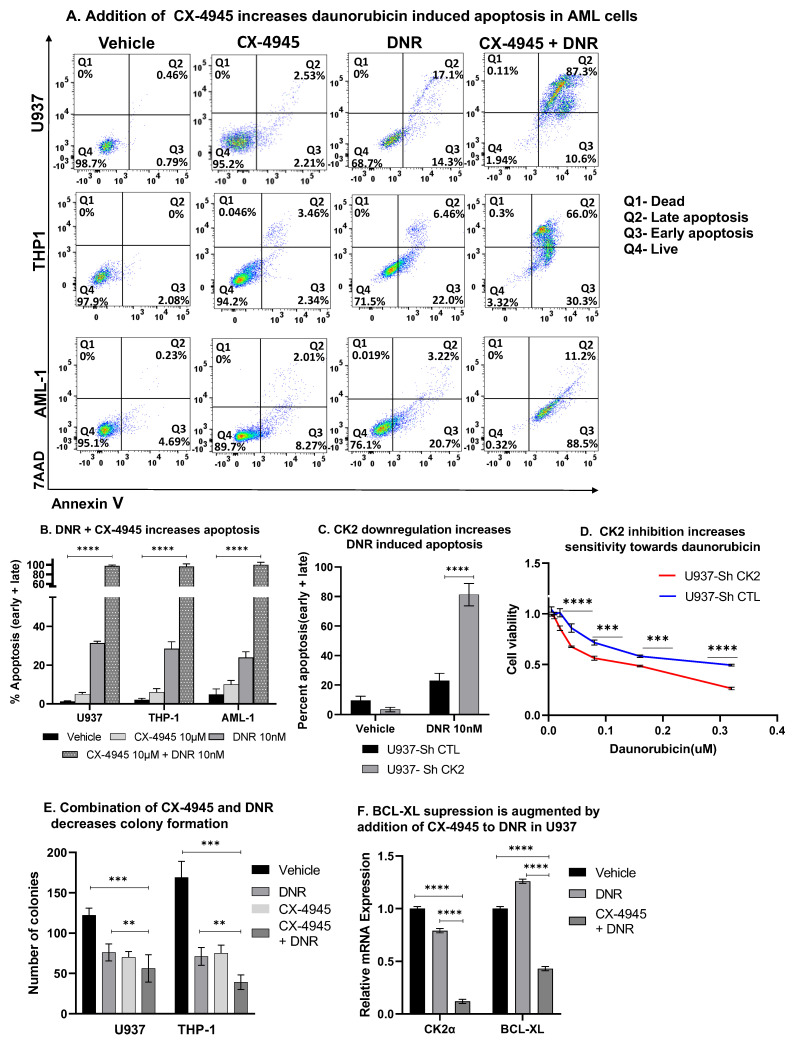
CK2 inhibition potentiates daunorubicin-induced apoptosis in AML cells. Cells were treated with 10 µM of CX-4945 or a combination of 10 µM CX-4945 with 10 nM of daunorubicin for up to 48 h. Cells were stained with 7-AAD and Annexin V for flow cytometry to assess apoptosis. (**A**) Flow plots showing representative results from three replicate experiments. The percentage of cells in the right upper and lower quadrant of each flow chart represents the percentage of late and early apoptotic cells, respectively, in U937 (top row), THP-1 (middle row), and AML-1 cells (bottom row) Q1: Dead, Q2: Late apoptosis, Q3: Early apoptosis, Q4: Live. Graphed in (**B**) are the mean +/−SD of triplicates from two independent experiments showing the percent of apoptosis cells following the drug treatment, as indicated above. (**C**) CK2α silencing in U937 cells achieved using ShCK2α and sorted for GFP after 24 h. Sorted cells were treated with 10 nM of DNR for 24 h before staining for Annexin V and 7AAD to assess apoptosis. The graph shows the combined percent apoptotic cells (early + late) in each group with and without the DNR treatment. (**D**) Cytotoxic drug response measured by the MTT assay after treating CK2α ShRNA treated U937 cells and the respective controls with various daunorubicin concentrations for 24 h. (**E**) Cells were pretreated as above for 48 h and were plated in a Methocult medium. Colonies were counted under an inverted light microscope. Colonies that contained around 50 cells or more were counted for analysis. Graphed in Figure 7E is the number of colonies after 14 days as the mean of three replicates +/− SD of two independent experiments. (**F**) The qRT-PCR showing decreases in the mRNA level in U937 cells treated with daunorubicin alone (10 nM) or a combination of CX-4945 and daunorubicin. *p*-value summaries are as follows: *p* > 0.05 (ns); *p* < 0.01 (**); *p* < 0.001 (***); *p* < 0.0001 (****).

The addition of CX-4945 potentiates DNR induced apoptosis (Figure 7A,B). Downregulation of CK2α resulted in increased apoptosis (Figure 7C) and decreased cell viability (Figure 7D) when exposed to DNR compared to the control cells. A combination treatment with CX-4945 and DNR showed decreased colony formation compared to the cells treated with DNR alone (Figure 7E). The repression of BCL-XL was significantly augmented following the treatment with CX-4945 and DNR compared to the DNR single-agent treatment (Figure 7F). A combination of CX-4945 and daunorubicin showed the synergetic cytotoxic activity in primary AML cells (Figure S9). Overall, these results provide evidence of the functional synergy between CX-4945 and daunorubicin in suppressing BCL-XL expression in AML.

## 4. Discussion

Protein kinase CK2 is essential for embryonic development, cell survival, growth, and maintenance [46,47]. The overexpression of CK2 enables cancer cells to proliferate and resist apoptosis [48,49,50]. Leukemia cells are more susceptible to CK2 inhibition-triggered cytotoxicity than their normal counterparts. CK2 has emerged as a potential anti-leukemia target [10,11,51]. CX-4945 is an orally bioavailable, ATP competitive, small molecule inhibitor that has shown a favorable toxicity profile and efficacy in patients with relapsed refractory cancers [22,24,52]. CX-4945 has shown strong in vivo efficacy in lymphoid leukemia when used as a single agent and combined with cytotoxic therapy [25,53,54]. In AML, several recent studies have shown cytotoxic activity of CX-4945 as a single agent and in combination with cytotoxic therapy using the in vitro cell system and murine leukemia models and describes another CX-4945 mechanism of action to induce cell death in AML [14,15,21,36,55]. Strategically targeting a promiscuous kinase CK2, which is indispensable for several physiological cellular pathways, is challenging [56]. This nature of CK2 underscores the need for a thorough investigation of all signaling transduction pathways affected by CK2 in different leukemias. A broad understanding of the mechanisms of action of CK2 inhibitors in AML helps design rational drug combinations that would be most effective to overcome chemoresistance, sensitize cells to cytotoxic therapy, and minimize off-target effects. 

In lymphoblastic leukemia, the tumor suppressor IKAROS is one of the important substrates of CK2 [37]. IKAROS (encoded by the *IKZF1* gene) is a master regulator of lymphoid hematopoiesis and a tumor suppressor in leukemia [57]. Unlike the B cell acute lymphoblastic leukemia (ALL), where more than 30% of cases have *IKZF1* genetic alterations, less than 10% of patients in AML have *IKZF1* genetic mutations [33]. The functional inactivation of IKAROS by CK2 hyperphosphorylation impairs IKAROS’s transcriptional regulatory functions in B cell ALL, independent of the genetic alteration [37]. 

Chromatin immunoprecipitation coupled with next-generation sequencing (ChIP-seq) was performed to assess IKAROS binding to DNA in U937 AML cells following the treatment with CX-4945. Results showed enhanced IKAROS binding peaks at the promoter region of several genes. IKAROS binding peaks were significantly increased at the promoter region of the *BCL-XL* (*BCL2L1*) gene. Further validation by the quantitative ChIP experiment showed that treating U937 and AML primary patient cells (AML-1) with CX-4945 enhances IKAROS binding to the *BCL-XL* gene. Functional experiments using the loss of function or gain of the IKAROS function have shown that IKAROS negatively regulates *BCL-XL* transcription via chromatin remodeling (formation of repressive chromatin). IKAROS is hyperphosphorylated in AML. The CK2 inhibition can reverse this process and restore the IKAROS regulation of *BCL-XL*. 

Here, we show that the forced overexpression of CK2 in AML cells led to increased cell proliferation, decreased apoptosis, and resistance to DNR. More importantly, CK2 overexpressing AML cells engrafted rapidly and decreased the survival time of mice. Downregulation and pharmacological inhibition of CK2 decreased the BCL-XL expression and sensitized the AML cells to DNR-induced apoptosis. Anthracyclines (DNR and mitoxantrone) are used extensively in the treatment of AML. These results provide a mechanistic basis for the use of CX-4945 in combination with DNR. 

This study shows that, in AML, IKAROS and CK2 play an essential role in the transcriptional regulation of the anti-apoptotic gene *BCL-XL*. Other anti-apoptotic genes regulated by CK2 and IKAROS include *BCL2A1* and *MCL-1*. Here, we focused on the transcriptional regulation of the anti-apoptotic gene *BCL-XL,* as this is one of the important genes regulating leukemia cell resistance to apoptosis [58]. The clinical use of BCL-XL inhibitors is limited due to the platelet destruction [59]. Here, we show that by targeting CK2, we can indirectly suppress BCL-XL expression by restoring the IKAROS transcription factor-mediated negative regulatory mechanism. Using two AML cell line (U937 and THP-1) derived xenograft models, we show that the treatment with oral CX-4945 causes decreased BCL-XL expression in vivo. Decreases in the BCL-XL expression correlated with decreases in the bone marrow engraftment suggesting an in vivo anti-leukemia effect. Interestingly, no decrease in the platelet count was noted in the CX-4945 treated AML xenograft mice (Table S4). Finally, the patient-derived AML xenograft mice treated with the CX-4945 single-agent oral therapy for 3 weeks showed significantly decreased leukemia burden and prolonged survival time compared to the vehicle-treated mice. Further preclinical studies are required to establish the in vivo efficacy of CX-4945 combined with chemotherapy such as daunorubicin in AML.

## 5. Conclusions

**Figure 8:**
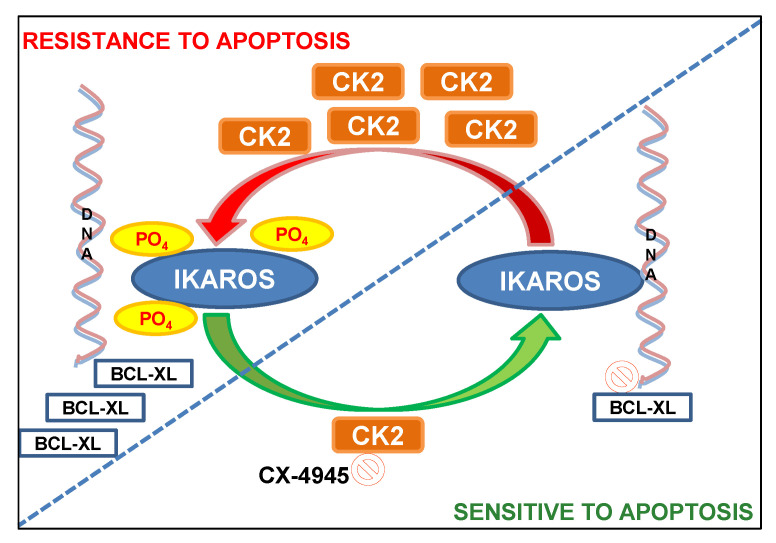
Model illustration of the regulation of apoptosis in AML by CK2 and IKAROS via repression of BCL-XL.

In summary, we report for the first time on the in vivo therapeutic efficacy of CX-4945 in AML patient-derived xenograft models. These results establish that one of the central mechanisms by which CX-4945 exerts an anti-leukemia effect in vivo is via the revival of IKAROS-mediated transcriptional repression of the *BCL-XL* gene (Figure 8). The functional synergy shown here, between the CK2 inhibitor and daunorubicin in suppressing BCL-XL provides a mechanistic basis for testing CK2 inhibitors combined with anthracycline therapy for the treatment of AML.

## Data Availability

The datasets analyzed and novel reagents used during the current study are available from the corresponding author upon request and after material transfer agreement. The ChIP-sequencing data shown in Figure 4 is available using the link provided in the Supplemental File.

## Supplementary Materials

The following are available online at https://www.mdpi.com/2072-6694/13/5/1127/s1, Figure S1: Western blot showing baseline protein overexpression in primary AML cells, Figure S2: Cell viability of U937, THP1 and AML#1 following CX-4945 treatment for 48 h, Figure S3: Western blot showing protein level of CK2α in U937 cells treated with CK2α sh-RNA(left panel), Figure S4: Immunoblot showing BCL-XL expression in CK2α overexpressing (OE) and silenced (CK2shRNA) U937 cells compared to control, Figure S5: Radioblot (top) and immunoblot (bottom panel) showing phosphorylated Ikaros in CK2α modulated U937 cells, Figure S6: Western blot showing CK2α and p-CK2 expression in U937 and THP-1 cells transduced with retroviral vec-tor containing CK2α, Figure S7: Histogram showing CK2α and BCL-XL protein quantification in U937 (left) and THP-1 (right) xenograft mice treated with CX-4945, Figure S8: Immunoblot showing Ikaros and BCL-XL protein expression in IKAROS overexpressed U937 cells, Figure S9: Synergetic cytotoxic effect of combination of Daunorubicin and CX-4945 in primary AML #1 cells, Table S1: List of Western blot antibodies, Table S2: the qChIP primers, Table S3: Characteristics of primary AML cells used in the study, Table S4: Complete blood count showing blood parameters in vehicle and CX-4945 treated U937 xenograft mice, Additional details of methods.
