# Supplementary material for: Mechanistic Basis for In Vivo Therapeutic Efficacy of CK2 Inhibitor CX-4945 in Acute Myeloid Leukemia
Source: Cancers (Basel). 2021 Mar 5;13(5):1127. doi: 10.3390/cancers13051127 (PMC7975325; doi:10.3390/cancers13051127)
Supplement: Supplementary file 1 [file cancers-13-01127-s001.zip › 7952-supply/cancers-1097952-supply.docx]

Supplementary Materials

Mechanistic Basis for in vivo Therapeutic Efficacy of CK2 Inhibitor CX-4945 in Acute Myeloid Leukemia

Morgann Klink, Mohammad Atiqur Rahman,Chunhua Song, Pavan Kumar Dhanyamraju,
Melanie Ehudin, Yali Ding, Sadie Steffen, Preeti Bhadauria, Soumya Iyer, Cesar Aliaga, Dhimant Desai,
Suming Huang, David Claxton, Arati Sharma and Chandrika Gowda


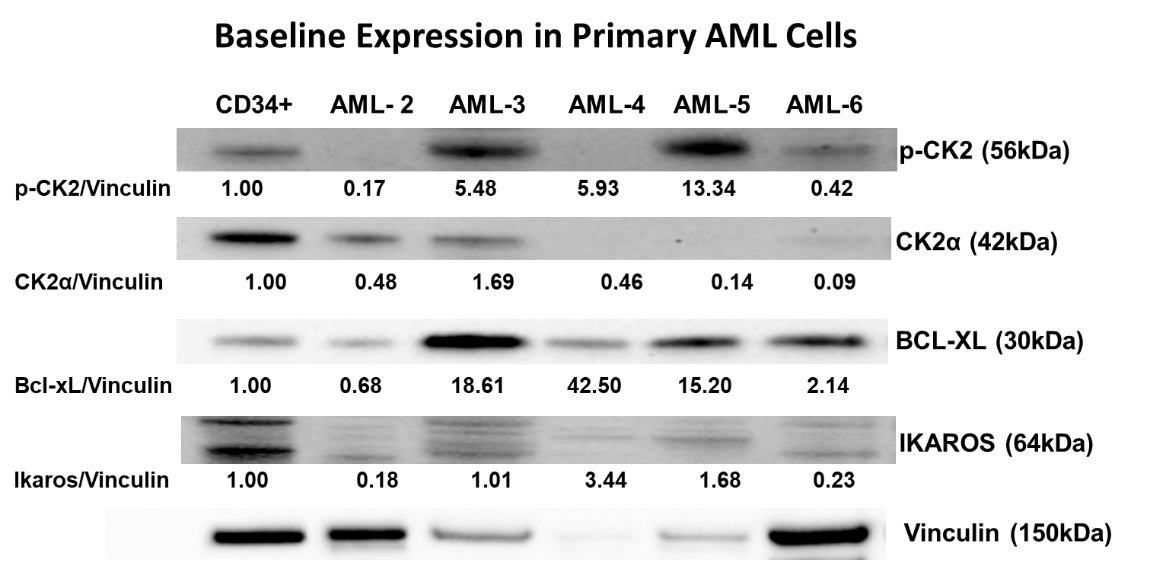


**Figure S1.** Western blot showing baseline protein overexpression in primary AML cells. UCB- Umbilical cord blood hematopoietic stem cells.

**Figure S2.** Cell viability of U937, THP1 and AML-1 following CX-4945 treatment for 48 hours.

**
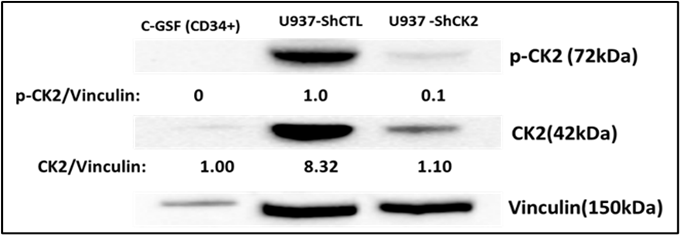

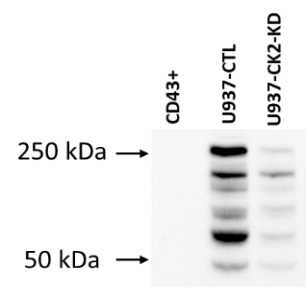

Figure S3.** Western blot showing protein level of CK2α in U937 cells treated with CK2α sh-RNA(left panel). Western blot showing decreased phosphorylated CK2 substrate protein levels in U937 CK2α knockdown cells.


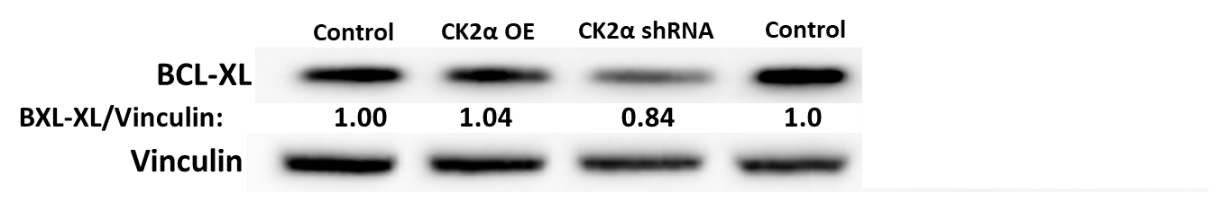


**Figure S4.** Immunoblot showing BCL-XL expression in CK2α overexpressing (OE) and silenced (CK2shRNA) U937 cells compared to control.


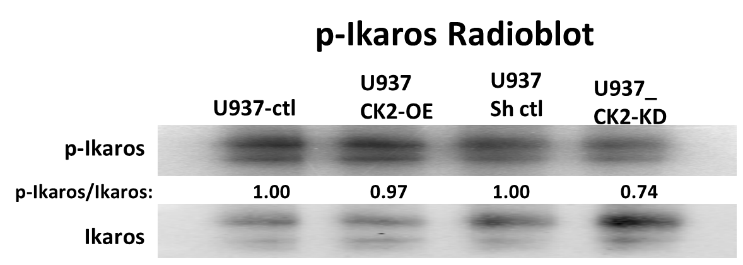


**Figure S5.** Radioblot (top) and immunoblot (bottom panel) showing phosphorylated Ikaros in CK2α modulated U937 cells.


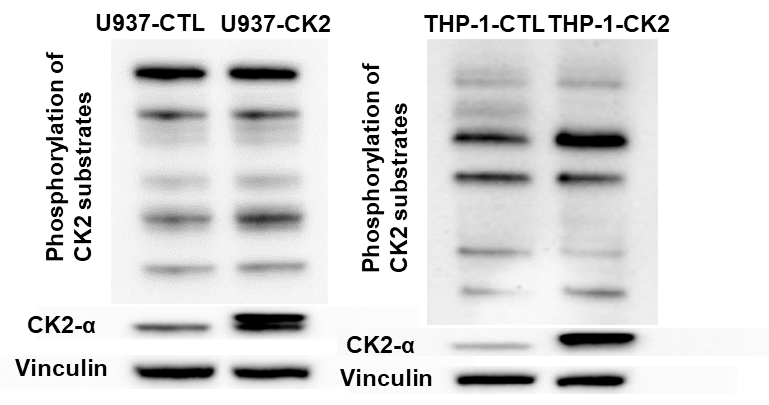


**Figure S6.** Western blot showing CK2α and p-CK2 expression in U937 and THP-1 cells transduced with retroviral vector containing CK2α.

**
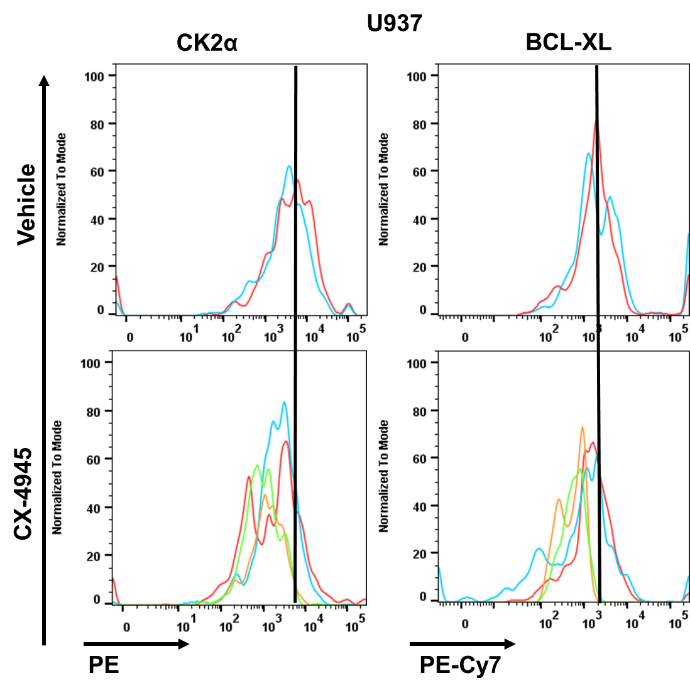

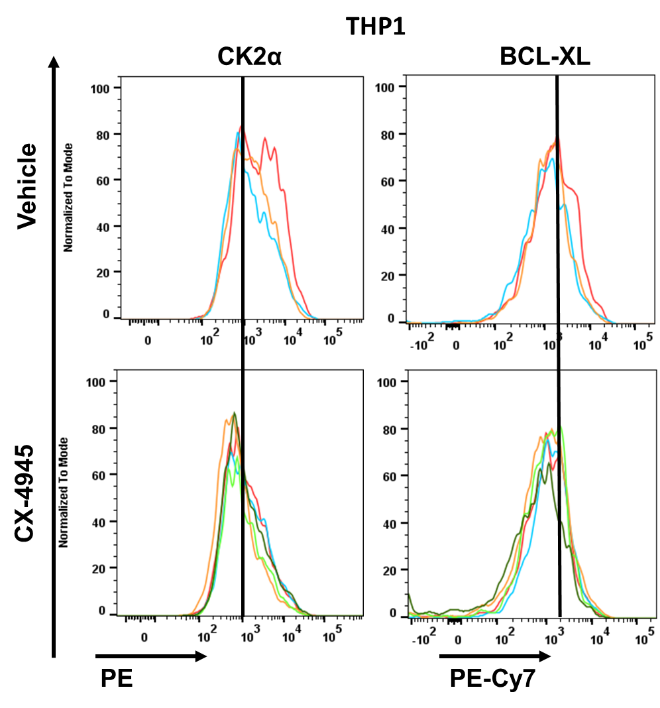
**

**Figure S7.** Histogram showing CK2α and BCL-XL protein quantification in U937 (left) and THP-1 (right) xenograft mice treated with CX-4945**.** Graph comparing the mean protein level is shown in Figure 3E.


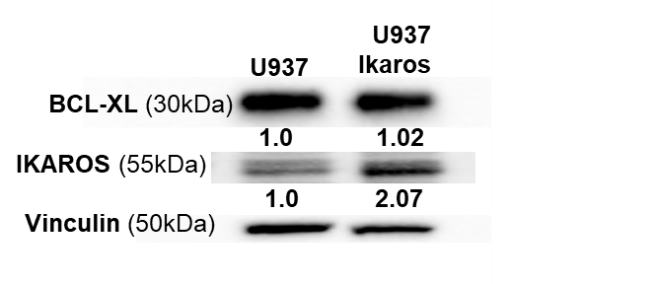


**Figure S8.** Immunoblot showing Ikaros and BCL-XL protein expression in IKAROS overexpressed U937 cells.


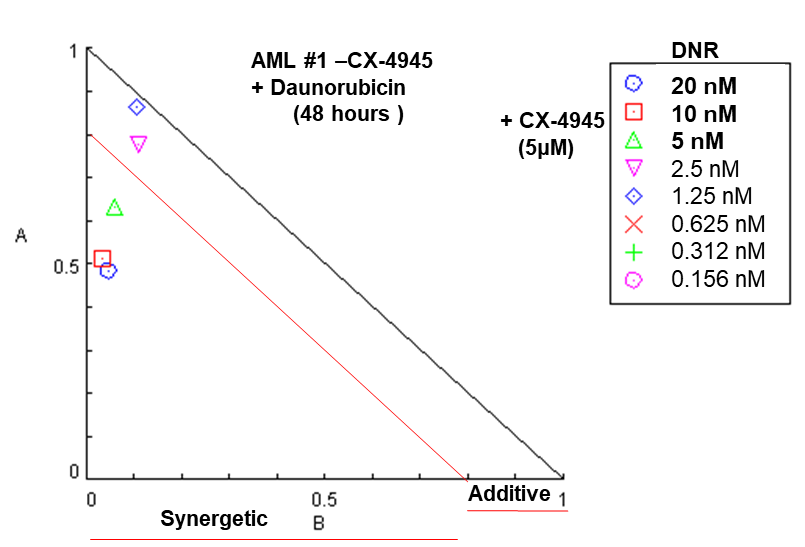


**Figure S9.** Synergetic cytotoxic effect of combination of Daunorubicin and CX-4945 in primary AML #1 cells. IC_50_ of CX-4945 is 3.79 μM. Synergetic effect noted with CX-4945 conc 5 μM with 5, 10 and 20 nM concentration of Daunorubicin over 48 hours.

**Table S1.** Immunoblot antibodies.

| **Name** | **Size** | **Catalog Number** | **Source** |
| --- | --- | --- | --- |
| BCL-XL | 30 kDa | 2764 | Cell Signaling Technology, Danvers, MA, USA |
| Phospho-CK2 Substrate [(pS/pT)DXE] MultiMab™ Rabbit mAb mix | Multiple proteins | 8738 | Cell Signaling Technology, Danvers, MA, USA |
| CK2α | 42 kDa | sc-373894 | Santa Cruz Biotechnology, Dallas, TX, USA |
| CK2α’ | 42 kDa | sc-514403 | Santa Cruz Biotechnology, Dallas, TX, USA |
| CK2β | 25 kDa | ab76025 | Abcam, Cambridge, MA, USA |
| p-AKT1 (S129) | 55 kDa | ab133458 | Abcam, Cambridge, MA, USA |
| AKT1 | 55 kDa | sc-5298 | Santa Cruz Biotechnology, Dallas, TX, USA |
| Vinculin | 150 kDa | 700062 | Sigma, St. Louis, MO, USA |
| IKAROS | 55-65 kDa | 66966-1-Ig | Proteintech, Rosemont, IL, USA |

**Table S2.** qChIP primers.

| **Kilobase** | **Forward** | **Sequence** | **Reverse** | **Sequence** |
| --- | --- | --- | --- | --- |
| -350 | BCL2L1-p-F | 5’- CCGGCAATCCCCAACTG-3’ | BCL2L1-p-R | 5’-CCGCTGGTTTGCTCTGAATT-3’ |
| 500 | BCL2L1-p1-F | 5’-GTGCTTTCGATTTGACTTAAG-3’ | BCL2L1-p1-R | 5’-CTCCAGGTACCAGAACTGGTTTC-3’ |
| 250 | BCL2L1-p2-F | 5’-CTCCTCTCCCGACCTGTGATAC-3’ | BCL2L1-p2-R | 5'-CACCACCTACATTCAAATC-3’ |
| -10 | BCL2L1-p3-F | 5’-GTGGCAGGAGGCCGCGGCTG-3’ | BCL2L1-p3-R | 5’- GAAGCTCAGGAACCAGCCCCCTC-3’ |
| -250 | BCL2L1-p4-F | 5’-GTCTCCGGCCTTCAACATC-3' | BCL2L1-p4-R | 5'-CAGTGAGGGACGCAGGGAG-3' |

**Table S3.** Characteristics of AML cells used in the study.

| **Cell Name** | **Characteristics** | | | | |
| --- | --- | --- | --- | --- | --- |
| CD34+ | Normal bone marrow hematopoietic stem cell | | | | |
| U937 | FAB M5 AML with t(10;11) | | | | |
| THP-1 | Infant AML with MLL rearrangement | | | | |
| K562 | chronic myeloid leukemia (CML) in blast crisis | | | | |
| **Primary AML Cells** | **Stage** | **Diagnosis** | **Cytogenetics** | **NPM1** | **FLT3** |
| AML1 | Diagnosis | AML-M4 | normal | WT (wild type) | WT |
| AML2 | Diagnosis | AML-M4 | normal | WT | ITD |
| AML3 | Diagnosis | AML-M4 | normal | WT | ITD |
| AML4 | Relapse | AML | complex | N/A | WT |
| AML5 | Diagnosis | AML-M4 | normal | mutant | WT |
| AML6 | Diagnosis | AML-M4 | normal | WT | WT |

ITD-Internal tandem duplicate.

**Table S4.** Complete blood count showing blood parameters in vehicle and CX-4945 treated U937 xenograft mice (n = 4).

| **Condition** | **WBC (10^3^/mm^3^)** | **RBC**  **(×10^6^/mm^3^)** | **Hgb (g/dl)** | **HCT**  **(vol. %)** | **MCV**  **(fl)** | **MCH (pg)** | **MCHC (g/dl)** | **Platelets (10^3^/mm^3^)** |
| --- | --- | --- | --- | --- | --- | --- | --- | --- |
| **Vehicle** | 2.0 ± 0.3 | 9.7 ± 0.8 | 16.0 ± 1.4 | 52.3 ± 3.6 | 53.9 ± 0.7 | 16.5 ± 0.1 | 30.6 ± 0.6 | 1333.7 ± 130.3 |
| **CX-4945** | 1.5 ± 0.3 | 9.8 ± 0.5 | 15.9 ± 0.7 | 52.5 ± 2.3 | 53.3 ± 0.5 | 16.2 ± 0.2 | 30.4 ± 0.3 | 1308.8 ± 152.3 |

Supplemental Methods

Plasmid Construction and Retroviral Gene Transfer

Wild-type human HA-tagged *IKZF1* cDNA was cloned by BglII and EcoRI site into the pMSCV bicistronic retroviral vector (MIG vector), which contains a 5' long-terminal-repeat-driven *IKZF1*, internal ribosome entry site (IRES), and enhanced green fluorescent protein (EGFP). Retroviruses were produced by transient transfection in amphotropic packaging HEK293T cell lines described previously [1–3]. According to the manufacturer's instructions, the lentivirus was concentrated by the Retro-XTM concentrator (Clontech Laboratories, Inc.). U937 cells were plated on 24-well plate at 4 × 10^5^ cells/well and suspended in retroviral supernatants with 12.5 µg/mL polybrene and centrifuged 1400 g, 32 °C, for 2 hours as previously described.[2] Cells were then suspended in fresh 10% FBS RPMI 1640 and cultured at 37 °C, in a 5% CO_2_ incubator for 3 days. The cells were Ficolled, and the GFP (+) cells were sorted with a FACSAria High-speed cell sorter (Becton Dickinson). The sorted cells were cultured for experiments.

IKZF1 and CK2α shRNA Knockdown

*IKZF1* knockdown was performed with lentiviral shRNA expression as described previously [1,4]. Briefly, U937 cells were infected with the lentivirus in a 24-well plate as described above. Seventy-two hours after infection, cells were sorted. The sorted cells are cultured for further biochemistry assays as detailed below. CK2α shRNA knockdown was performed using the Neon Transfection System (Invitrogen) as previously reported. We tested 4 CK2α shRNAs generated by cloning shRNA into a lentiviral vector with GFP as we described in the methods, all shRNA constructs were confirmed by sequencing, and the effect of the lentivirus on the CK2α knockdown was validated by qPCR. Two of 4 shRNAs showed a potent knockdown of CK2α mRNA level as measured by qPCR (one is shown in Figure 3A). Treatment with either CK2α shRNA resulted in upregulation of the BCL-XL gene.

*IKZF1*shRNA knockdown was also done with this method. Briefly, 29mer shRNA constructs for human *IKZF1* and human CK2α (CSNK2A1) in a GFP vector (pGFP-V-RS) were purchased from Origene (Rockville, MD, USA). U937 cells were transiently transfected with the shRNA plasmids or scramble shRNA control using the transfection system. After transfection for 1 day, U937 cells with transfection efficiency ranges from ~80% (green cells), and more than 95% cell viability was further treated with 10 µM CX-4945 or vehicle control (0.01% DMSO) for 2 days and harvested for total RNA isolation and total lysate extraction. The 29-mer scrambled shRNA cassette in pGFP-V-RS vector was also used as a control. Knockdown of *IKZF1* and CK2 was confirmed by qRT-PCR measurement of *IKZF1* mRNA levels and western blot measurement of IKAROS protein levels using anti-IKAROS-CTS antibody as reported previously. Two of four shRNAs showed a strong knockdown of *IKZF1* mRNA level as measured by qPCR, and one of them is showed in the figure.

ChIP-seq assays for IKAROS in U937 cells treated with CX-4945 (10 µM for 72 hours) cells were performed as reported previously [1,5]. Global analysis of IKAROS ChIP seq in CX-4945 treated U937 will be made available on the Gene Expression Omnibus (GEO) once manuscript is published. Link for accessing the data presented in Figure 4 is provided below.

<https://genome.ucsc.edu/cgi-bin/hgTracks?db=hg19&lastVirtModeType=default&lastVirtModeExtraState=&virtModeType=default&virtMode=0&nonVirtPosition=&position=chr1%3A150547027%2D150552214&hgsid=994685925_Ls2m4bFxupyQFtfcAsMrk2sRaxXA>

Briefly, ChIP-seq assays for IKAROS in U937 cells were performed as reported previously[6]. For the ChIP-seq library, affinity-purified anti-IKAROS antibody [3] was incubated with chromatin. ChIP-seq DNA sample preparation kit (Illumina) was used to create ChIP-seq libraries and 200–400-bp fraction was extracted and purified. Libraries were sequenced and ChIP-seq sequences were generated by an Illumina Hiseq 2000 or Genome Analyzer II system and then were mapped onto HG19 (Human Genome version 19 from NCBI) using the ELAND algorithm. CisGenome version 2.0 was used to detect binding peaks on HG19. Parameters remained at default settings for the program run.

Quantitative Chromatin Immunoprecipitation Assay

IKAROS qChIP assays ware performed as described previously [2,6]. Chromatin was incubated in buffer (1% triton X-100, 0.1% deoxycholate, 1× TE and protease inhibitor) with 10 µg affinity-purified rabbit polyclonal anti-IKAROS antibody or normal rabbit IgG (Abcam, ab46540) as a control which was pre-coated onto Goat-anti-rabbit IgG Dyneabeads (Invitrogen) overnight at 4 °C. The protein/DNA complexes were captured with Magnetic Particle Concentrator (Invitrogen), extensively washed with RIPA buffer (50 mM Hepes pH 8.0, 1 mM EDTA pH 8.0, 1% NP-40, 0.7% deoxycholate, 0.5 M LiCl). DNA was eluted with 50 µL elution buffer (10 mM Tris pH 8.0, 1 mM EDTA, 1% SDS). The eluates were de-crosslinked, treated with proteinase K, extracted with phenol/chloroform and RNase A. DNA was recovered using the QIAquick PCR Purification kit (QIAGEN). Enrichment of the ChIP sample over input (fold enrichment) was evaluated by qPCR with three or more replicates, using specific primers in the promoter region of *BCL2L1/BCL-XL* gene. Primers are shown in Supplemental Table 3. Histone modification markers qChIP assay were done with the same protocol as IKAROS qChIP by incubating chromatin with anti-H3K927me3 , anti H3K4me3 (Millipore, 07-449) or H3K9ac (Abcam, Ab4441) antibody or normal rabbit IgG (Abcam, ab46540) as a control.

References

1. Song, C.; Gowda, C.; Pan, X.; Ding, Y.; Tong, Y.; Tan, B.H.; Wang, H.; Muthusami, S.; Ge, Z.; Sachdev, M., et al. Targeting casein kinase II restores Ikaros tumor suppressor activity and demonstrates therapeutic efficacy in high-risk leukemia. *Blood* **2015**, *126*, 1813–1822, doi:10.1182/blood-2015-06-651505.
2. Song, C.; Ge, Z.; Ding, Y.; Tan, B.H.; Desai, D.; Gowda, K.; Amin, S.G.; Gowda, R.; Robertson, G.; Yue, F., et al. IKAROS and CK2 regulate expression of BCL-XL and chemosensitivity inhigh-risk B-cell acute lymphoblastic leukemia. *Blood* **2020**, doi:10.1182/blood.2019002655.
3. Gurel, Z.; Ronni, T.; Ho, S.; Kuchar, J.; Payne, K.J.; Turk, C.W.; Dovat, S. Recruitment of ikaros to pericentromeric heterochromatin is regulated by phosphorylation. *J Biol Chem* **2008**, *283*, 8291–8300, doi:10.1074/jbc.M707906200.
4. Gowda, C.; Song, C.; Ding, Y.; Iyer, S.; Dhanyamraju, P.K.; McGrath, M.; Bamme, Y.; Soliman, M.; Kane, S.; Payne, J.L., et al. Cellular signaling and epigenetic regulation of gene expression in leukemia. *Adv Biol Regul* **2020**, *75*, 100665, doi:10.1016/j.jbior.2019.100665.
5. Wang, H.; Song, C.; Ding, Y.; Pan, X.; Ge, Z.; Tan, B.H.; Gowda, C.; Sachdev, M.; Muthusami, S.; Ouyang, H., et al. Transcriptional Regulation of JARID1B/KDM5B Histone Demethylase by Ikaros, Histone Deacteylase 1 (HDAC1), and Casein Kinase 2 (CK2) in B Cell Acute Lymphoblastic Leukemia. *J Biol Chem* **2015**, doi:10.1074/jbc.M115.679332.

6. Song, C.; Pan, X.; Ge, Z.; Gowda, C.; Ding, Y.; Li, H.; Li, Z.; Yochum, G.; Muschen, M.; Li, Q., et al. Epigenetic regulation of gene expression by Ikaros, HDAC1 and Casein Kinase II in leukemia. *Leukemia* **2016**, *30*, 1436–1440, doi:10.1038/leu.2015.331.

| 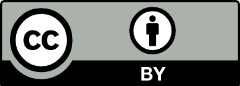 | © 2021 by the authors. Licensee MDPI, Basel, Switzerland. This article is an open access article distributed under the terms and conditions of the Creative Commons Attribution (CC BY) license (http://creativecommons.org/licenses/by/4.0/). |
| --- | --- |
