# Supplementary figures and images for: Mechanistic Basis for In Vivo Therapeutic Efficacy of CK2 Inhibitor CX-4945 in Acute Myeloid Leukemia
Source: Cancers (Basel). 2021 Mar 5;13(5):1127. doi: 10.3390/cancers13051127 (PMC7975325; doi:10.3390/cancers13051127)

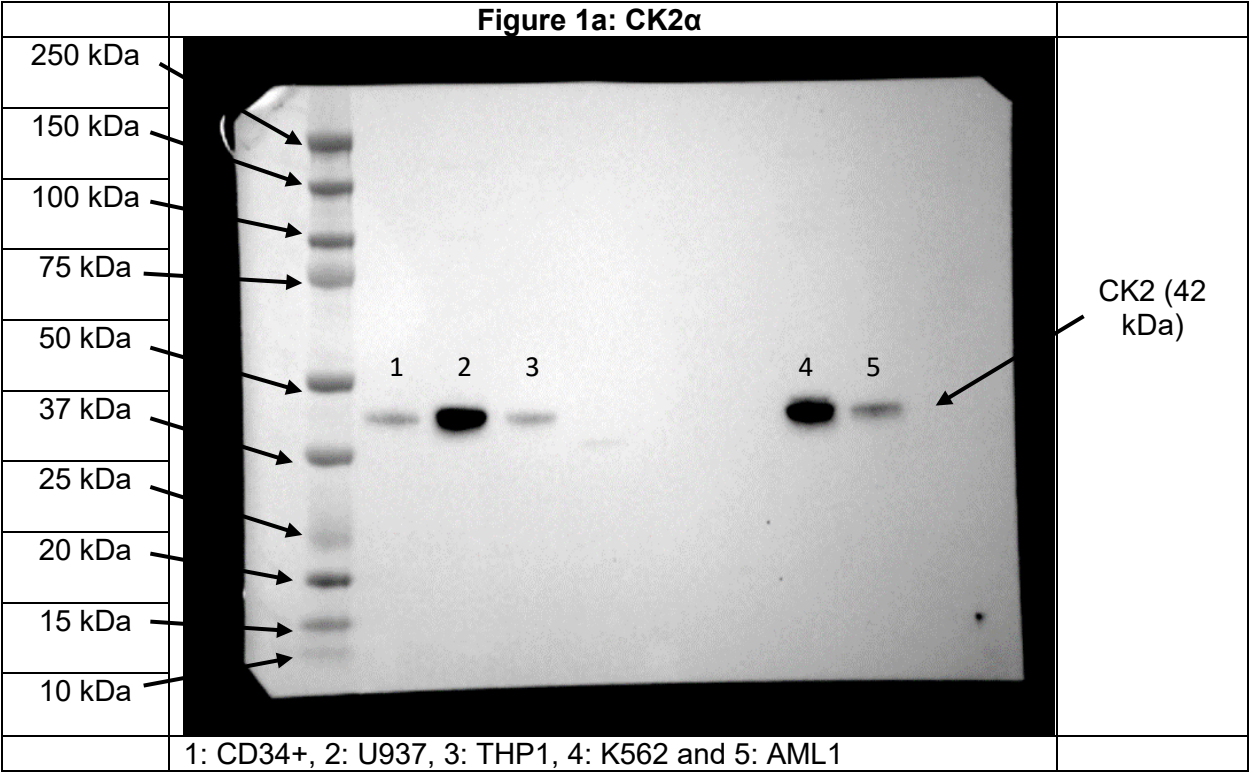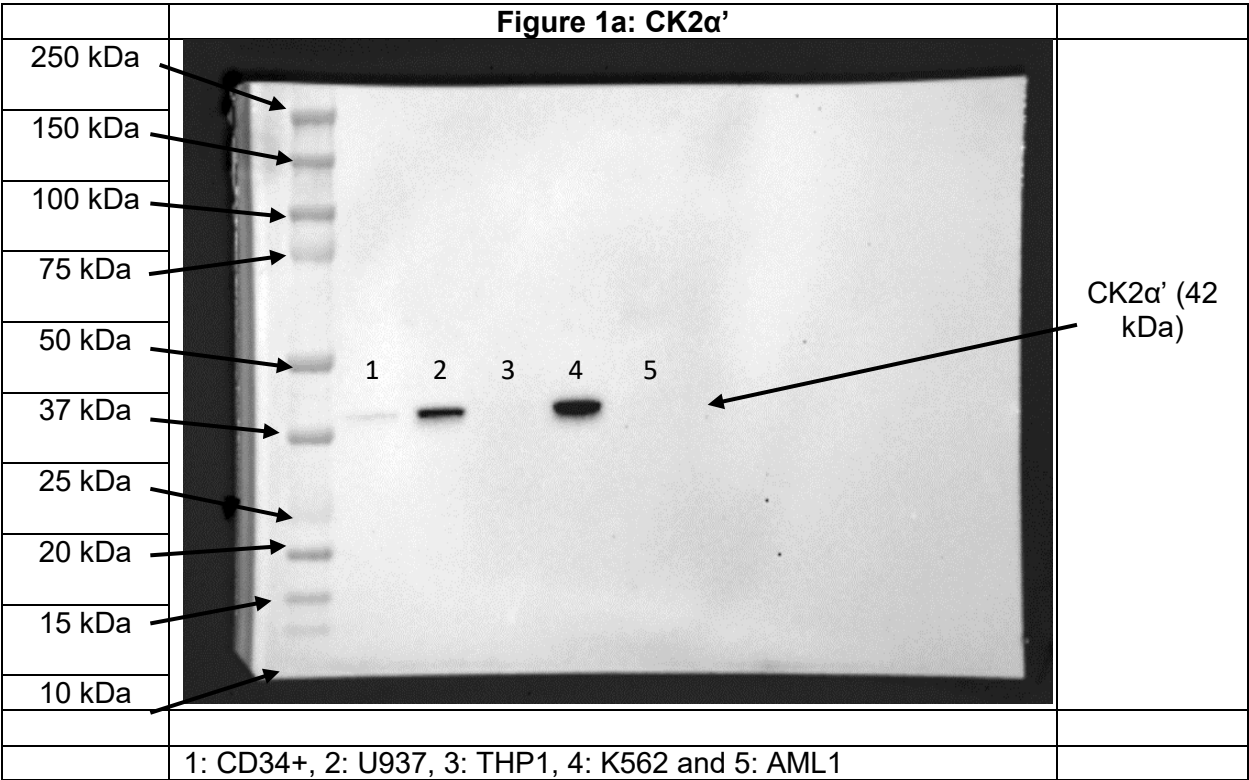

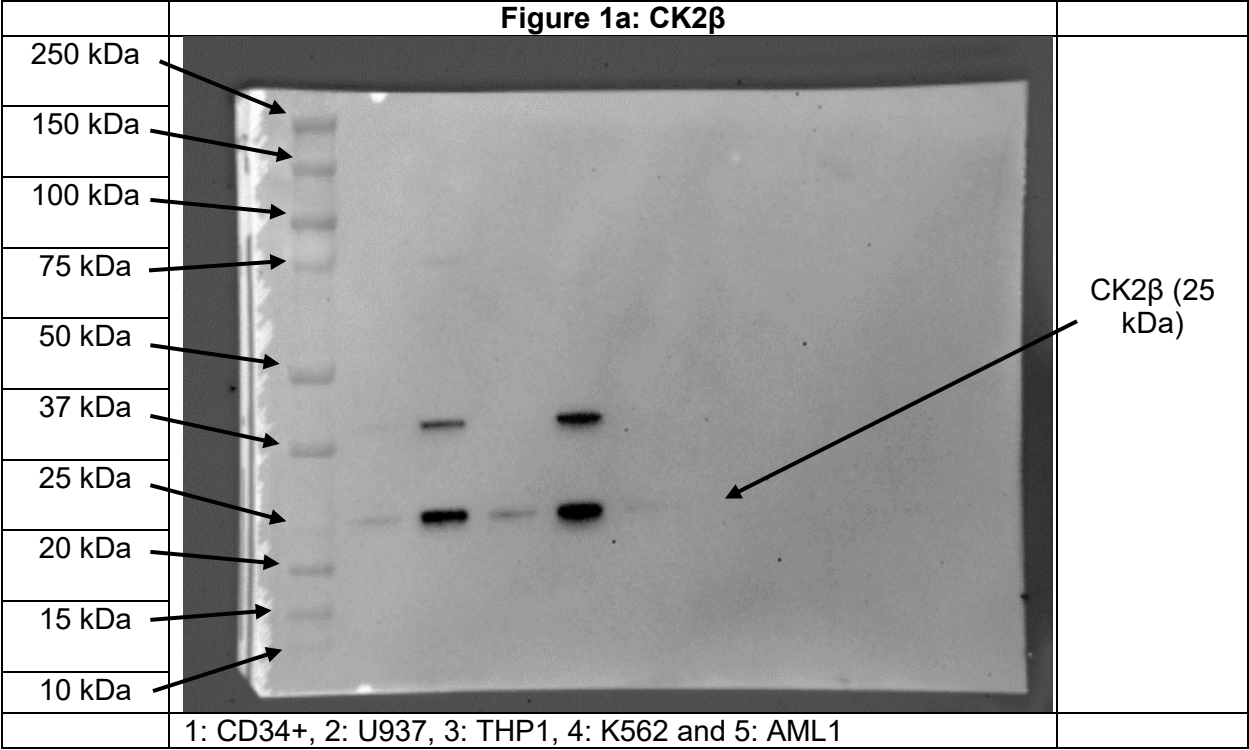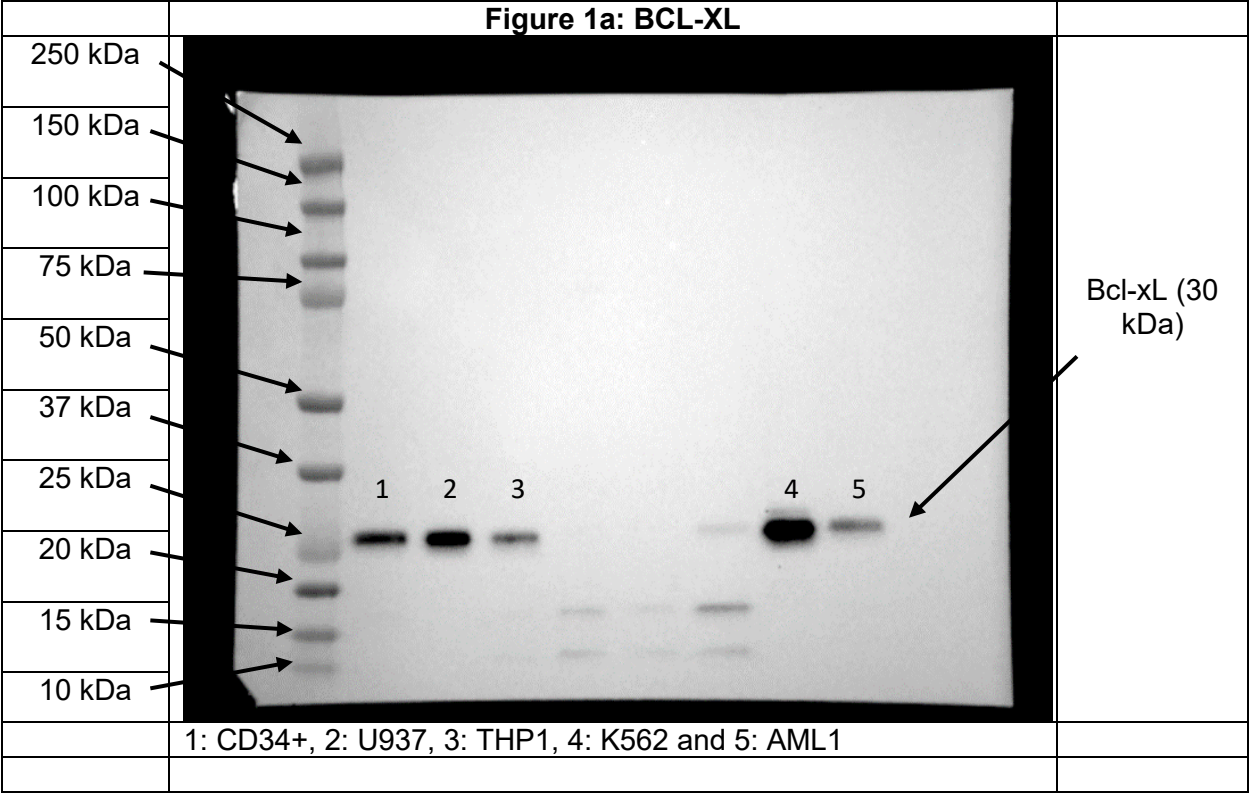

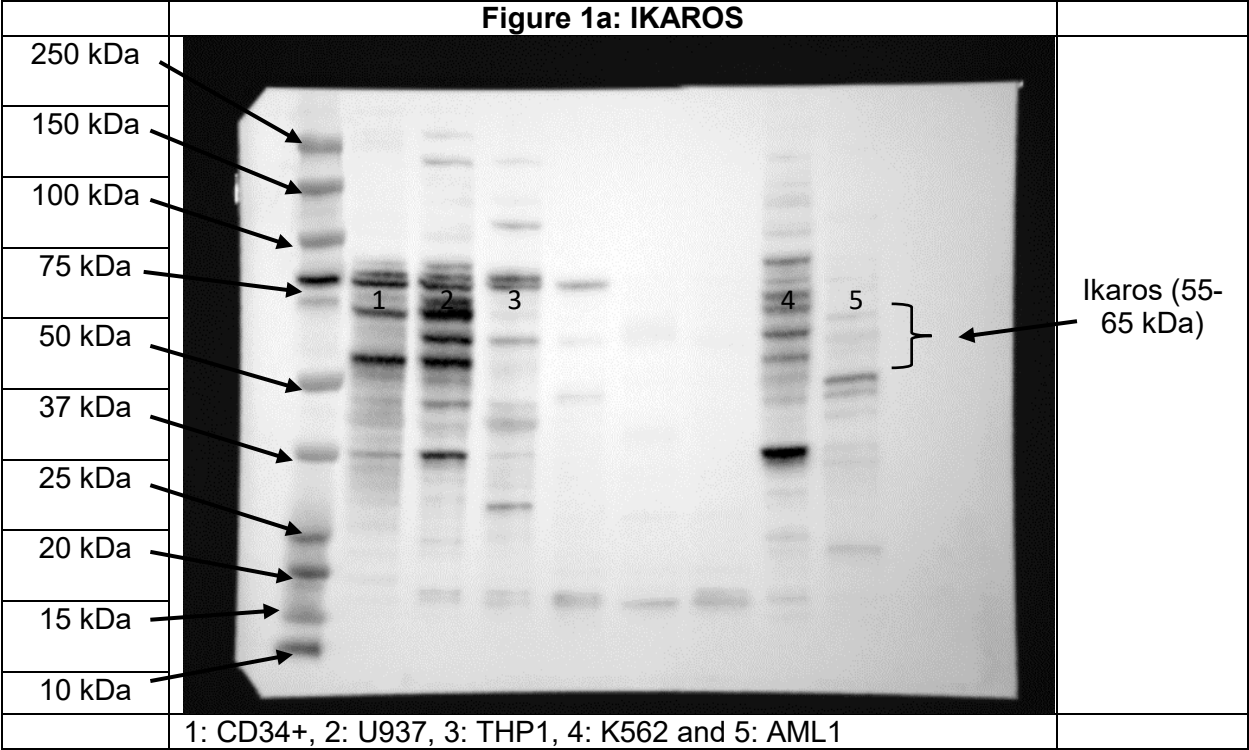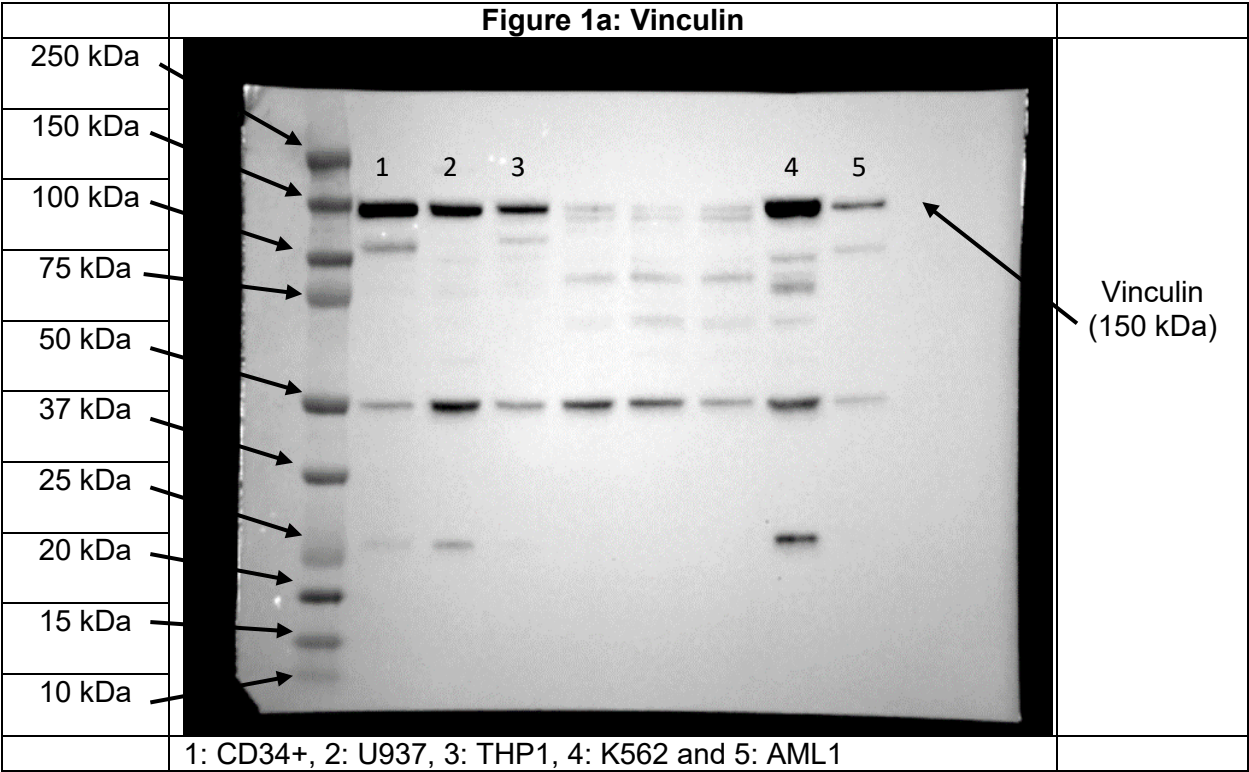

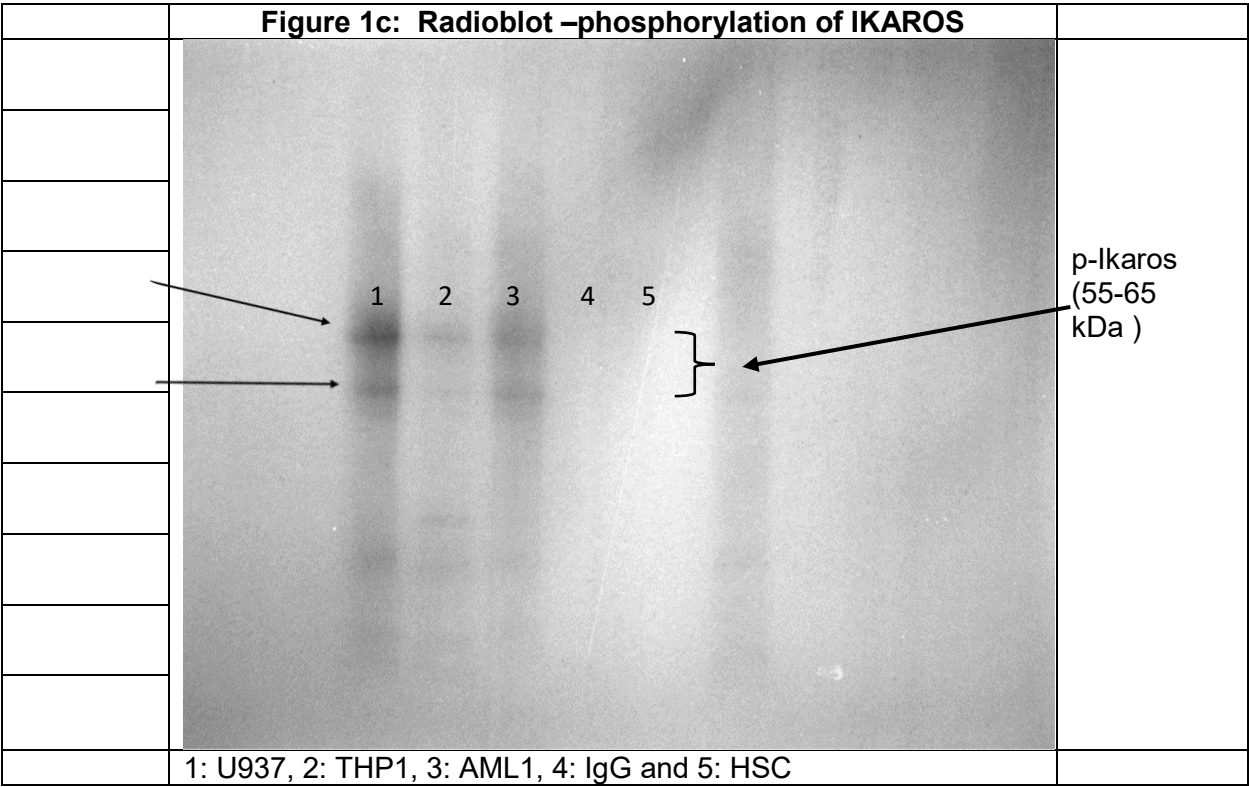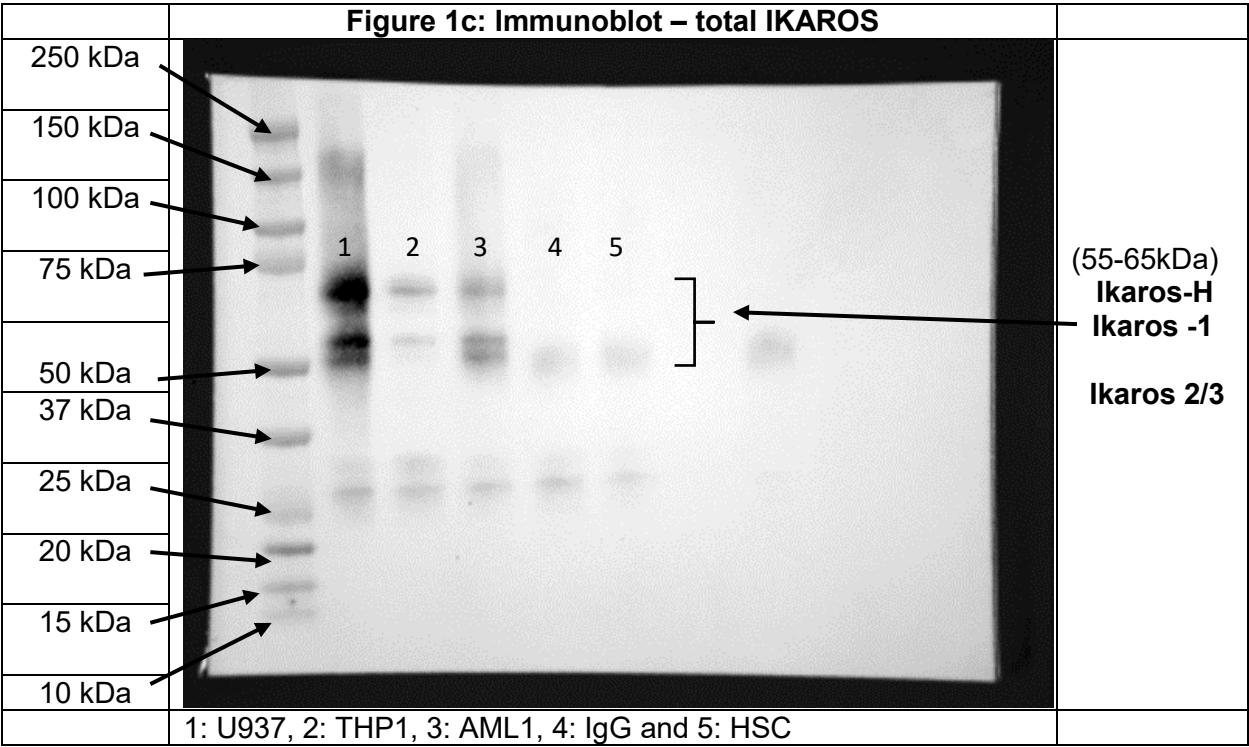

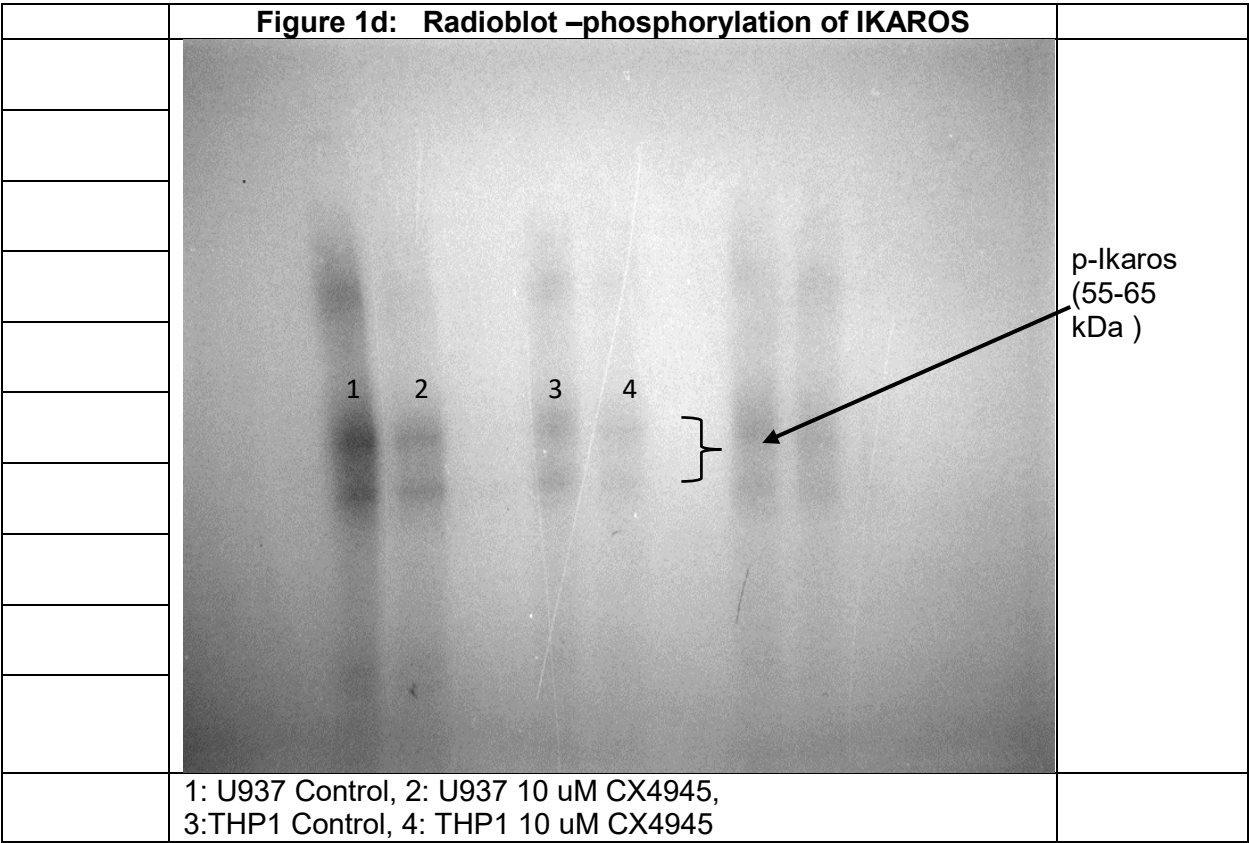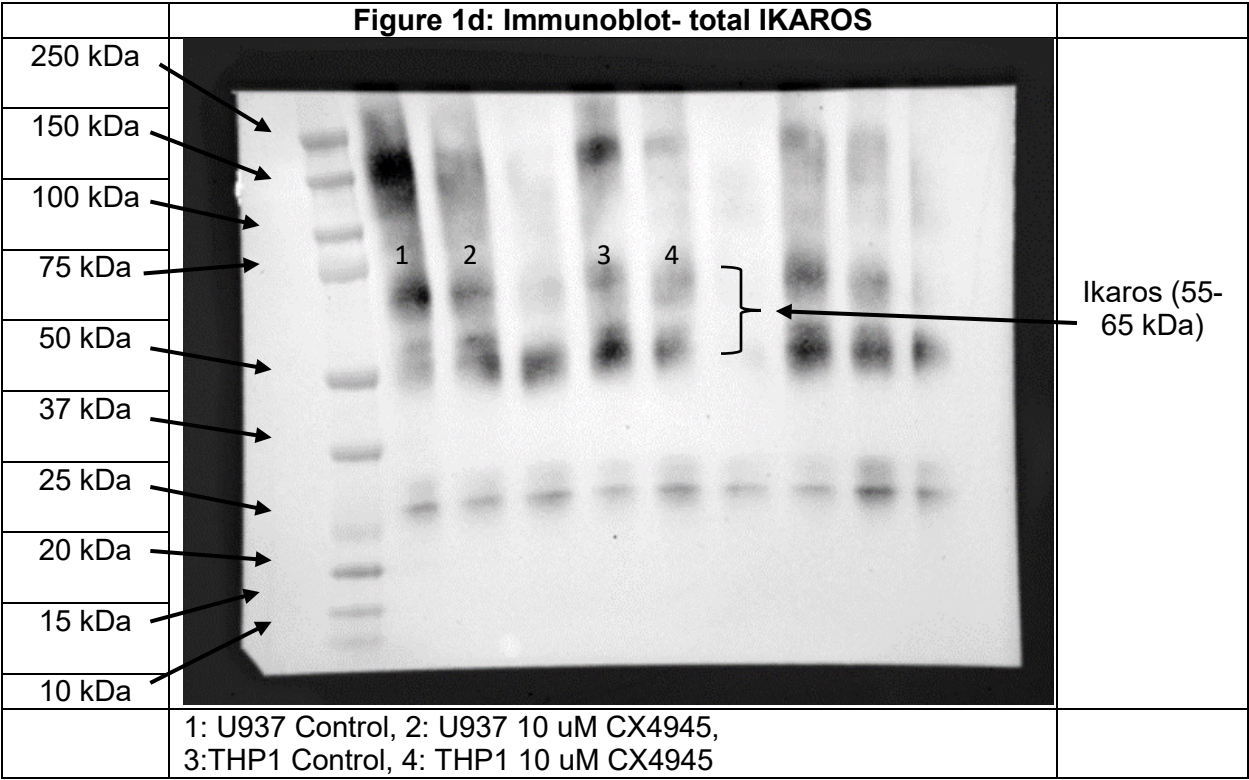

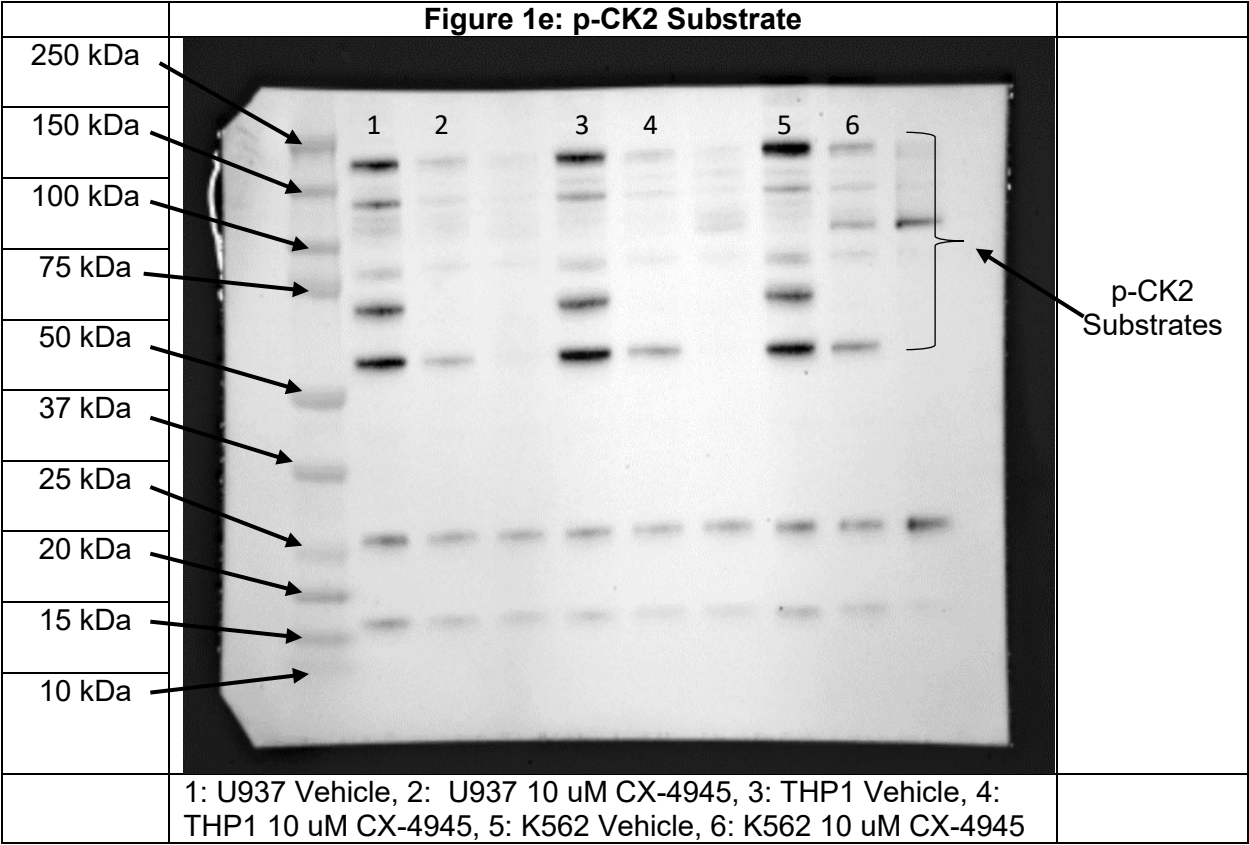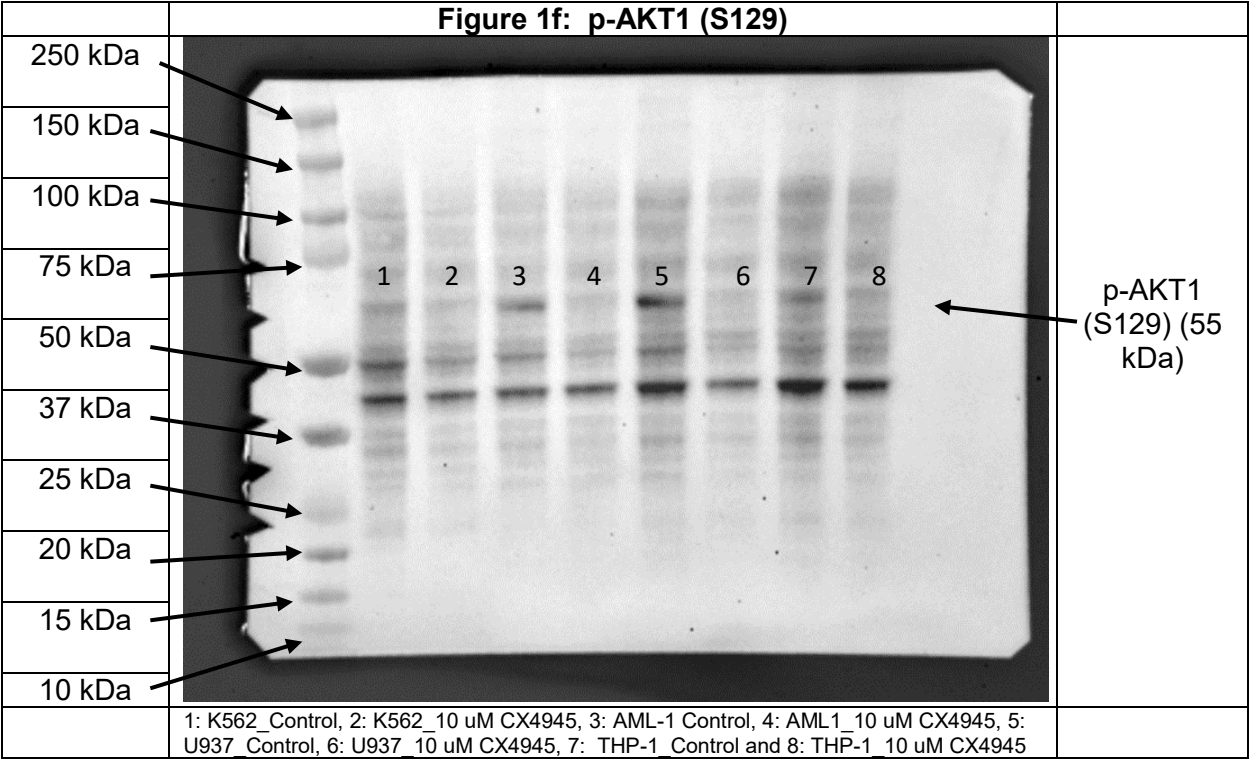

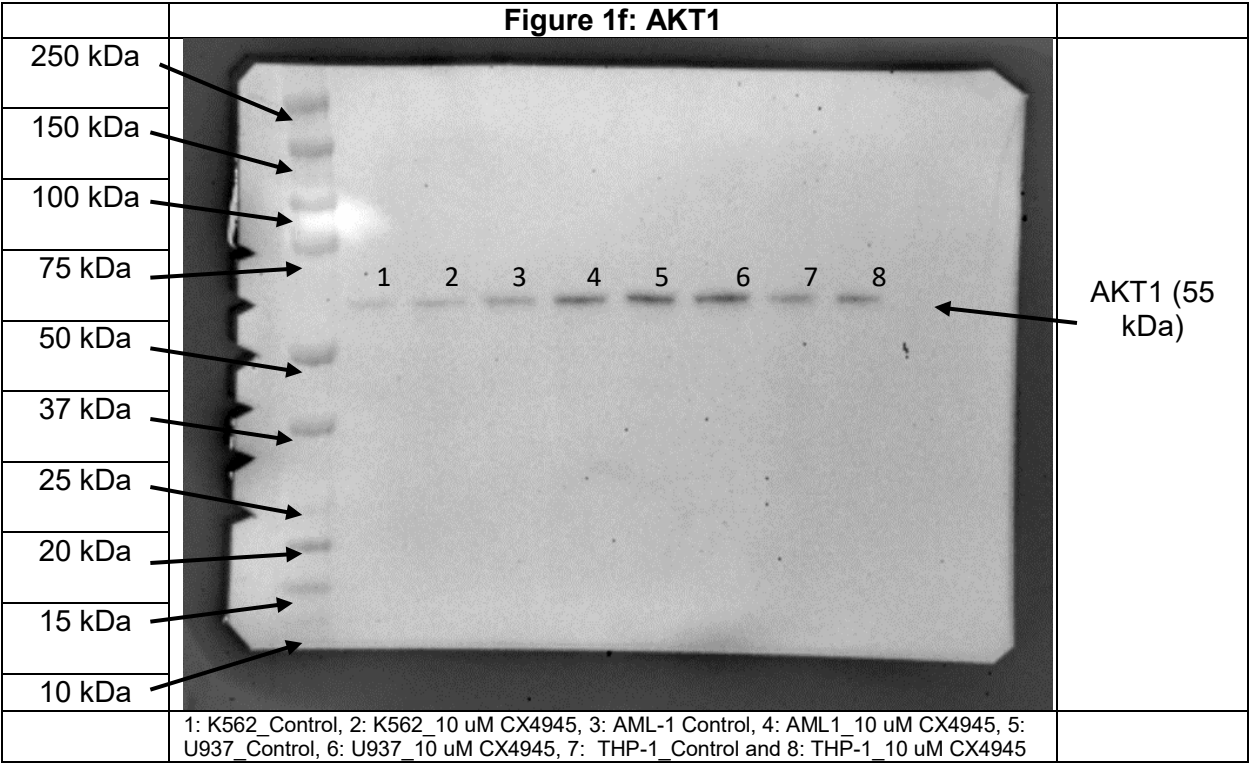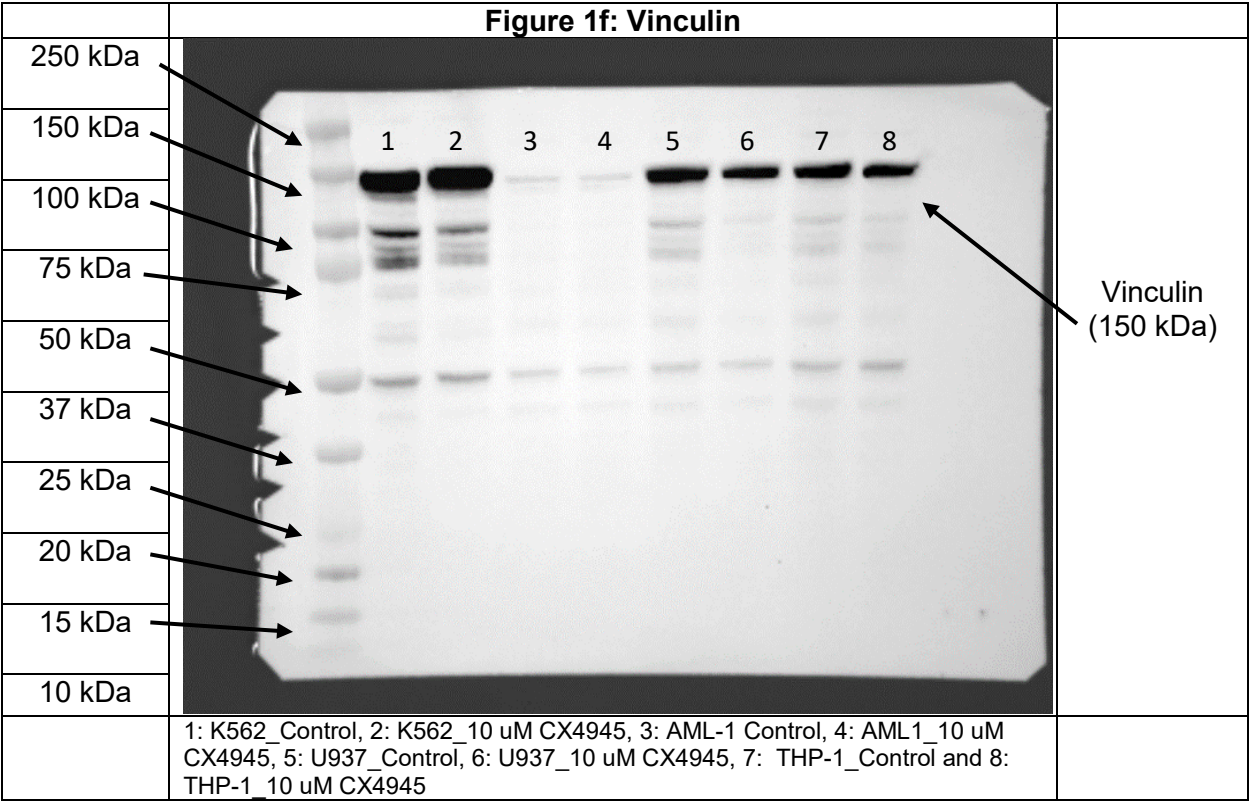

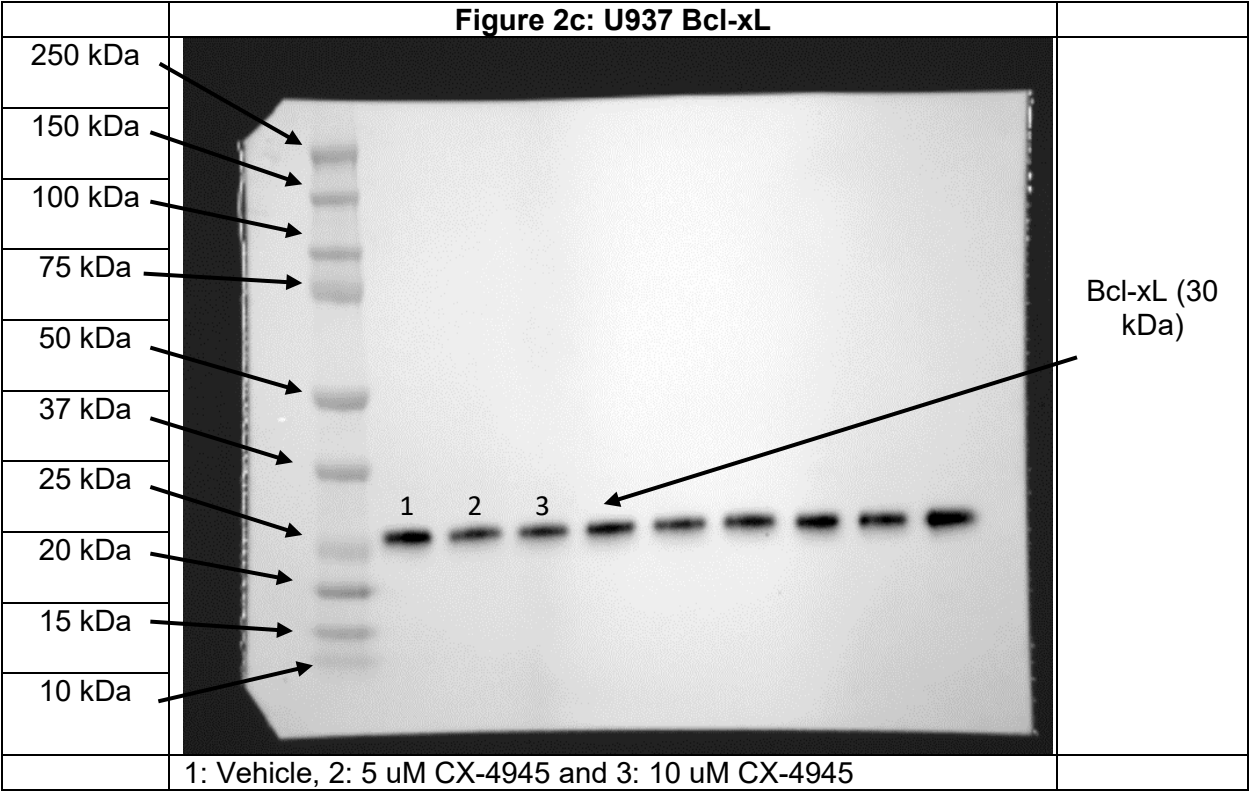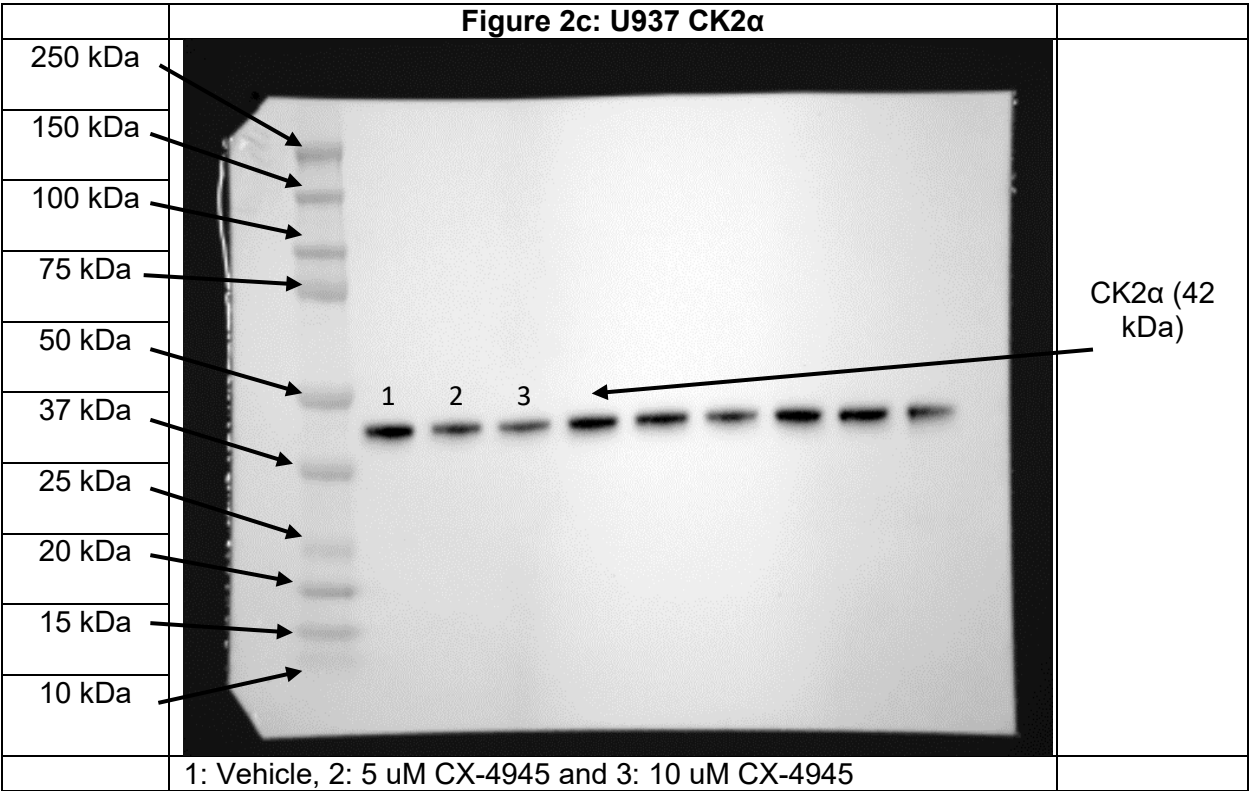

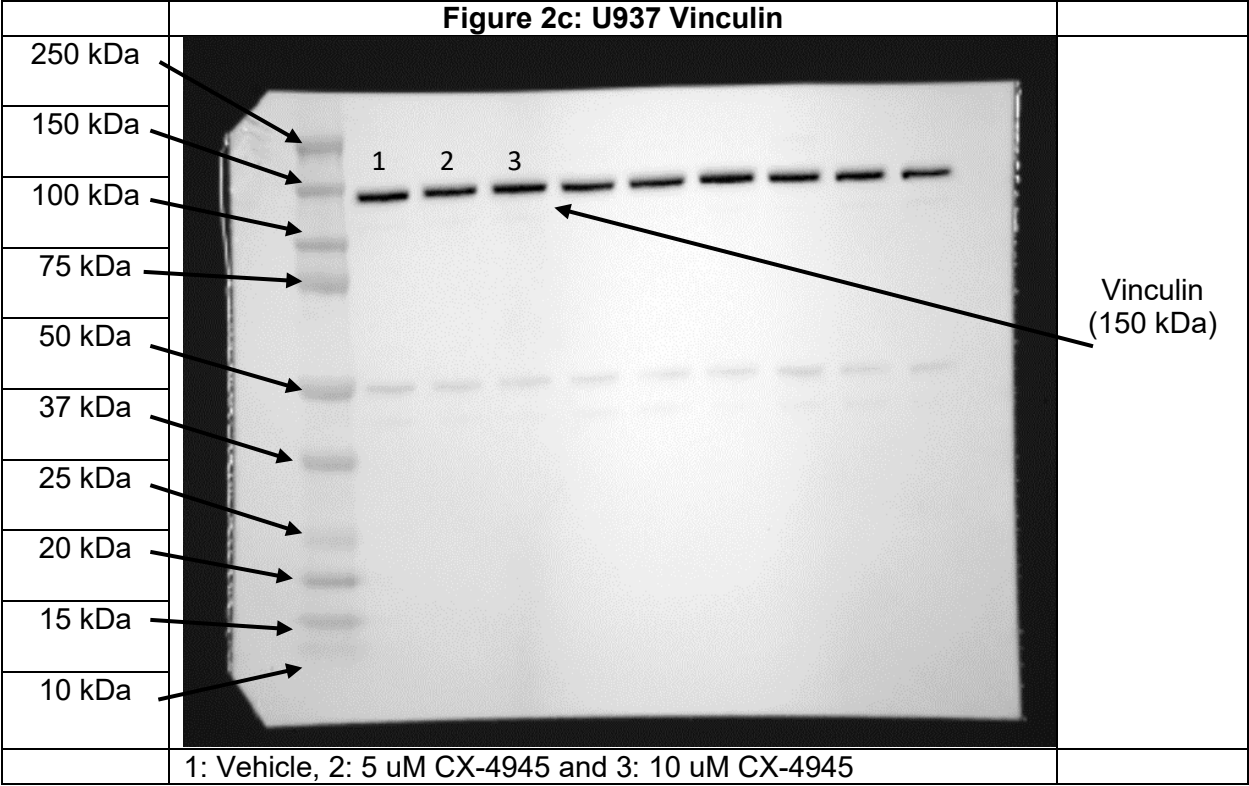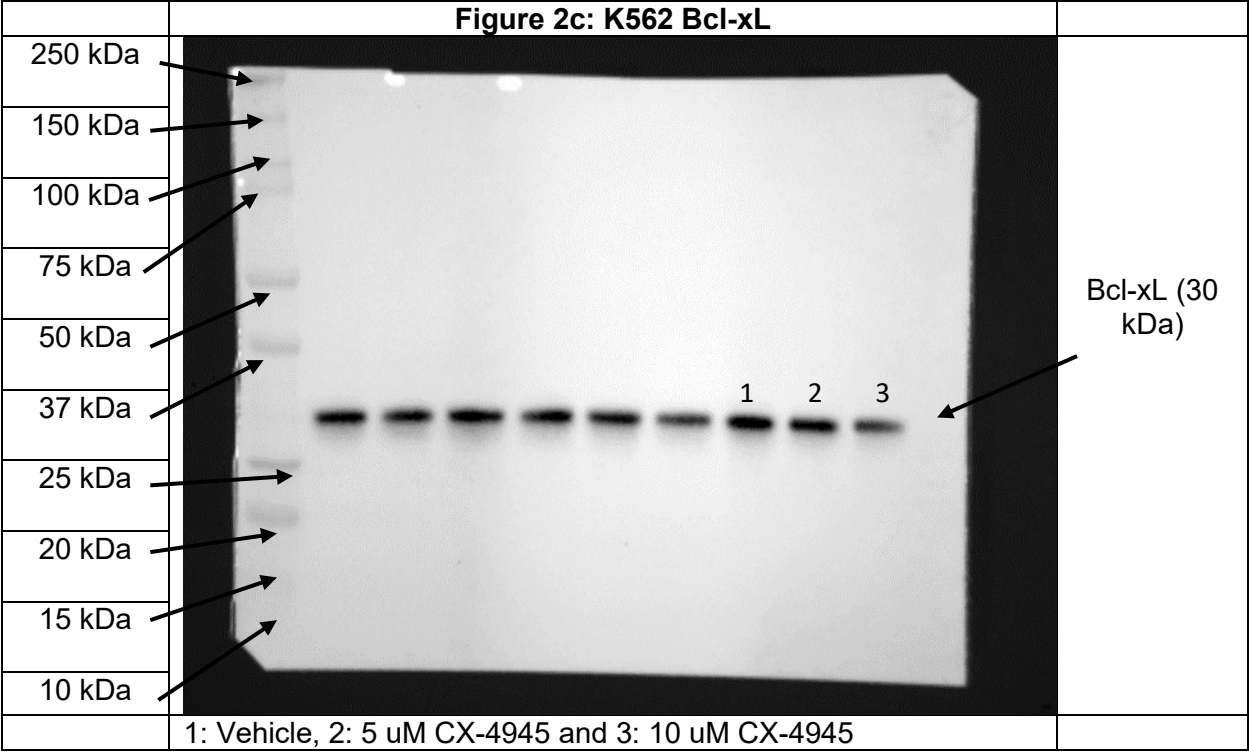

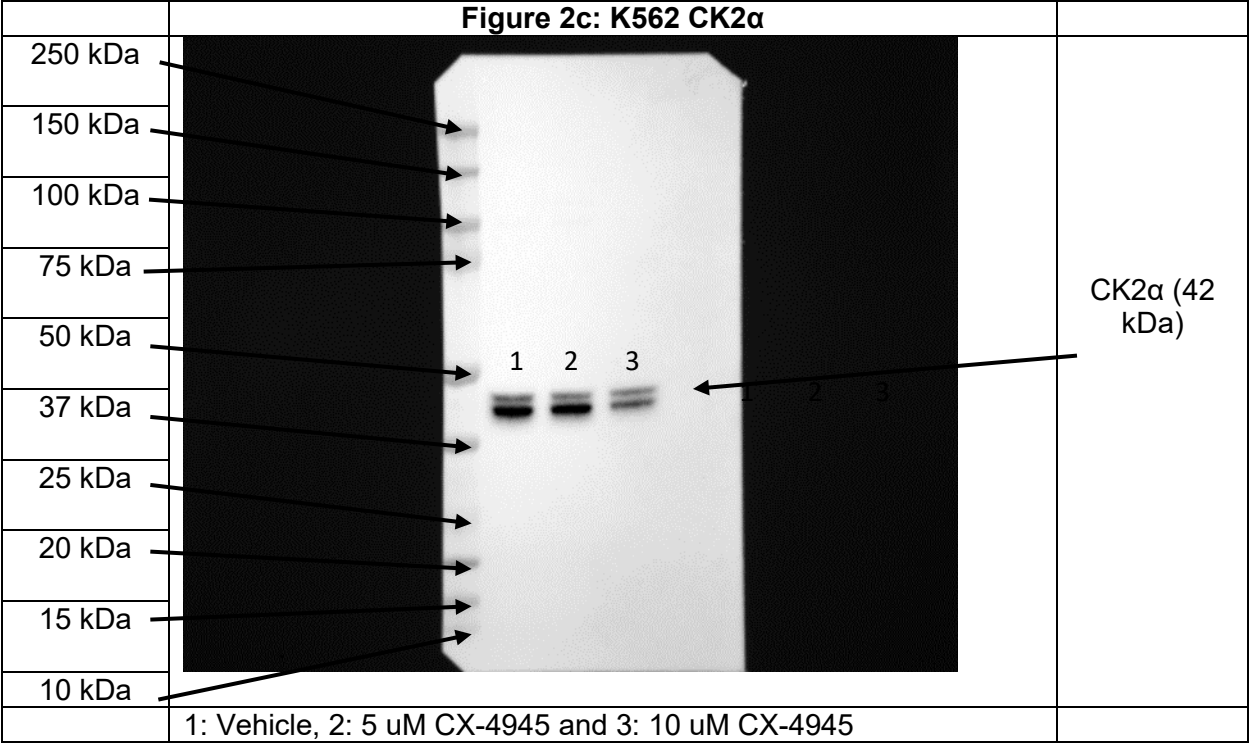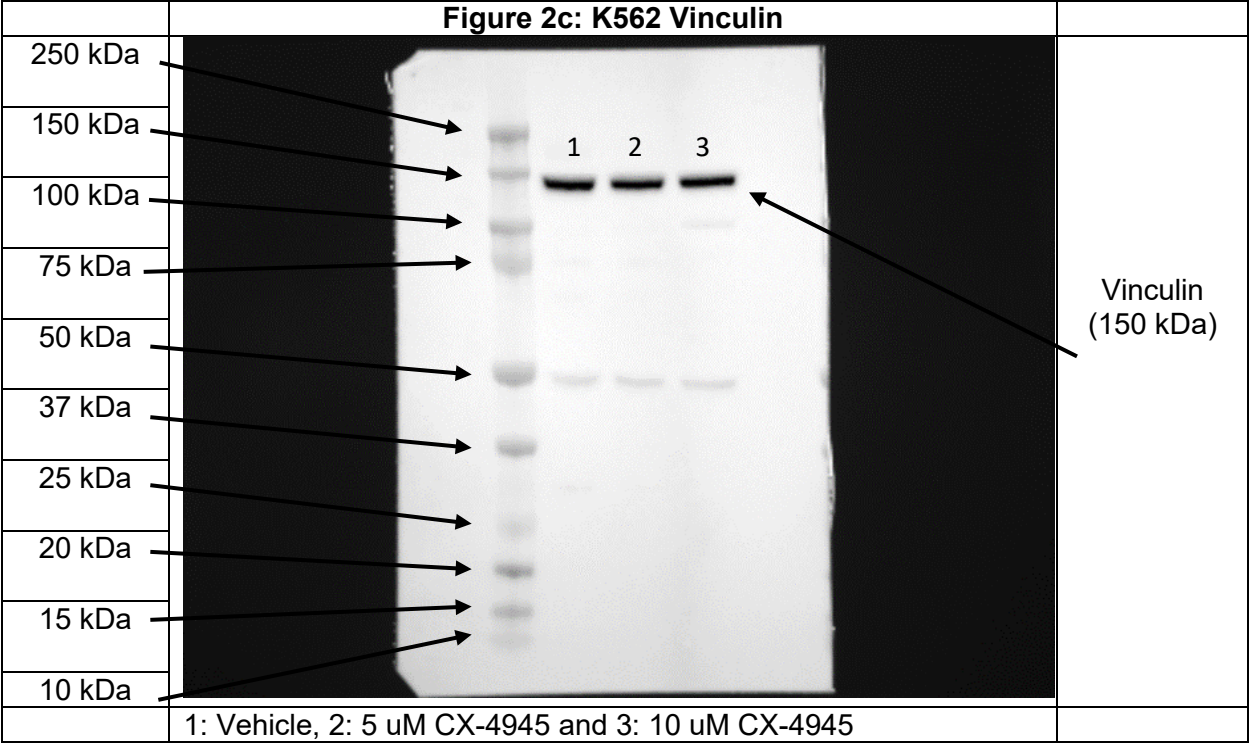

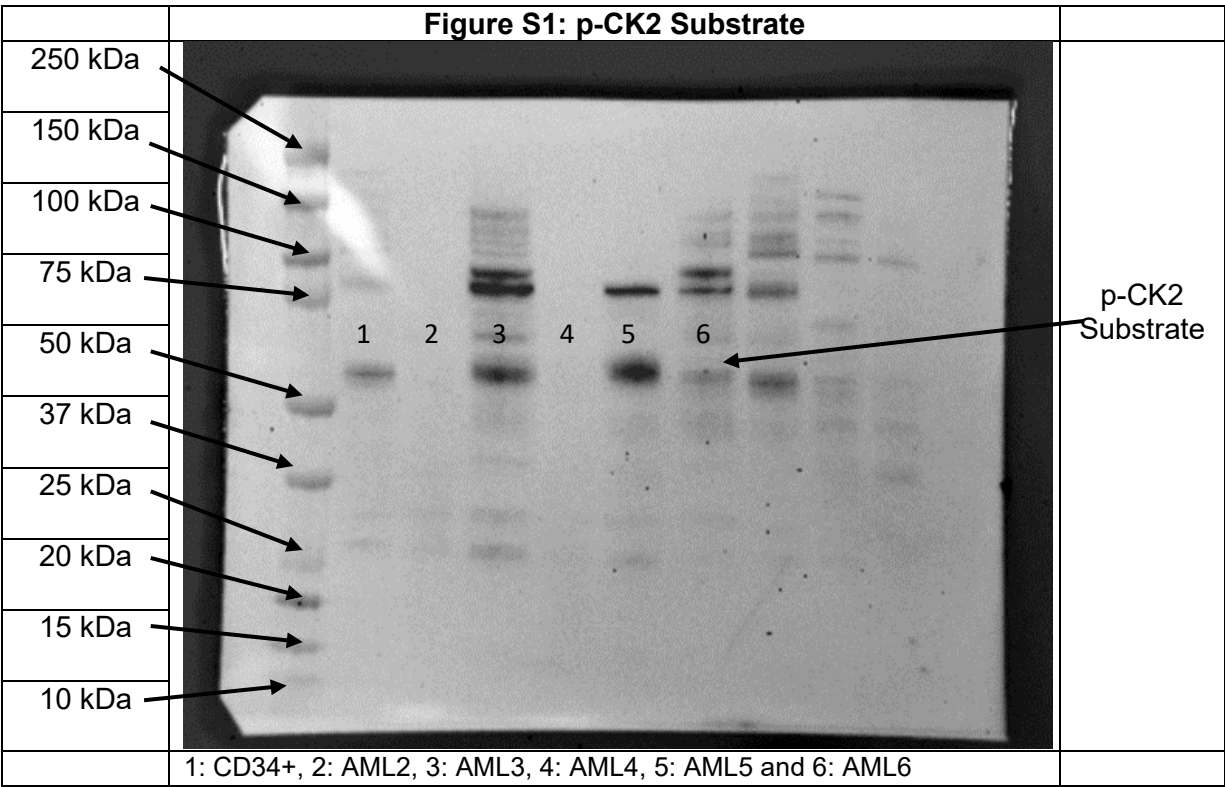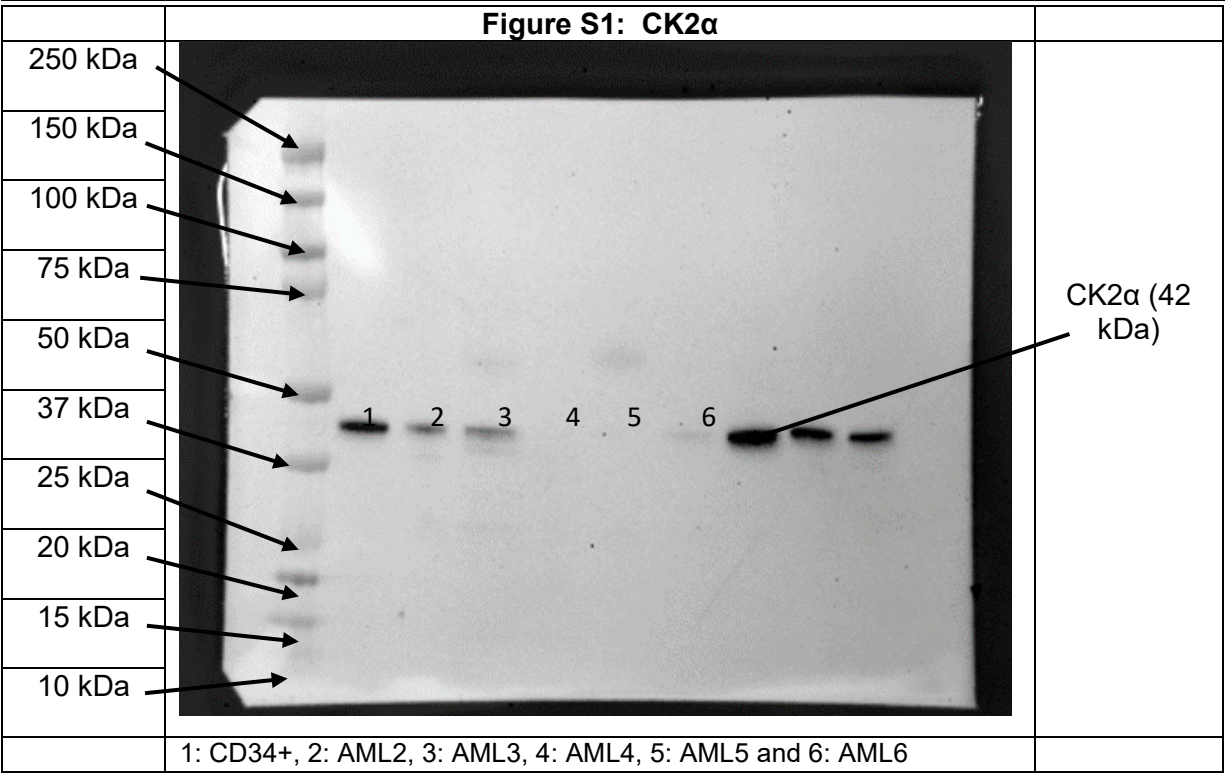

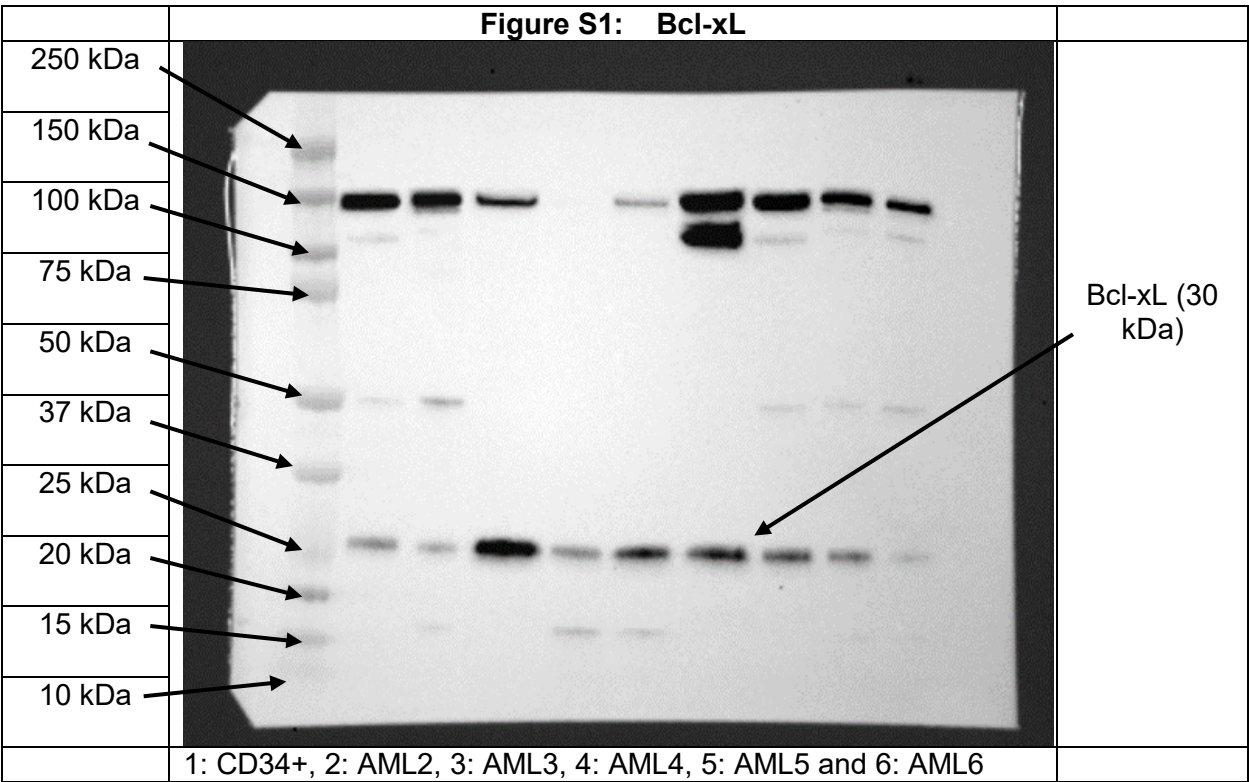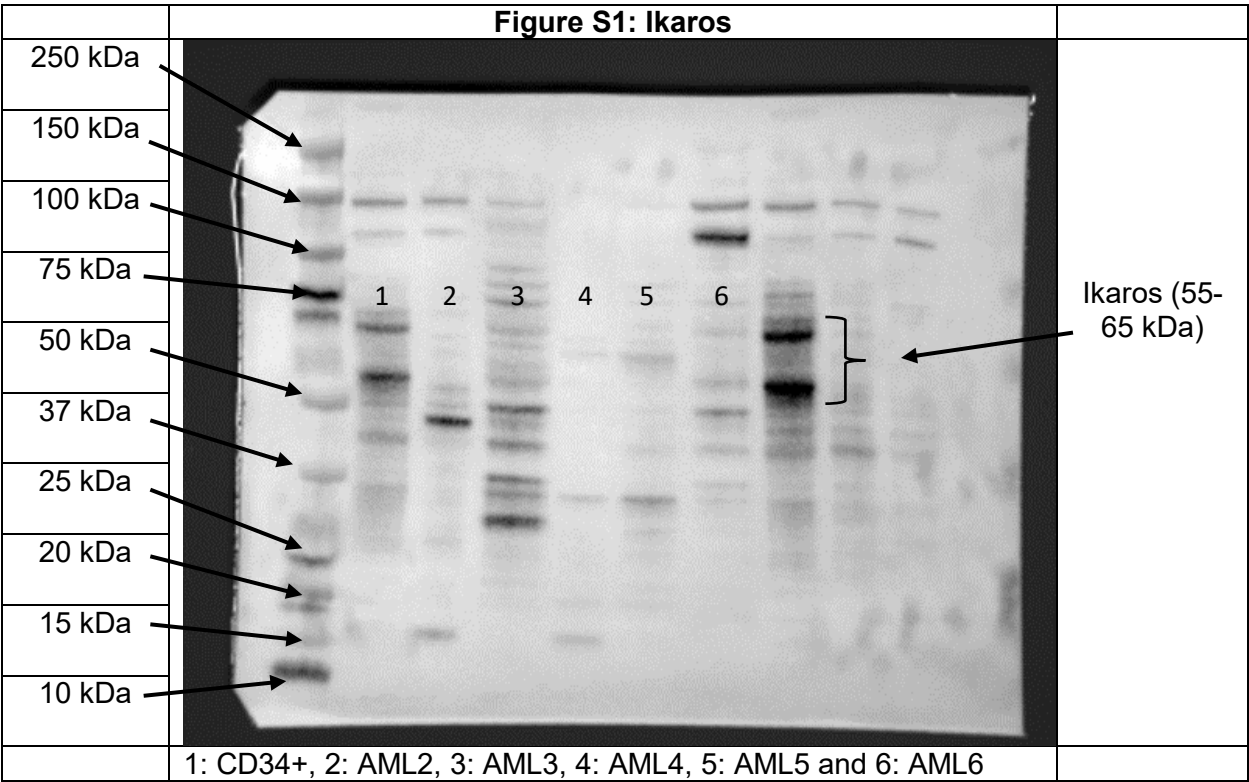

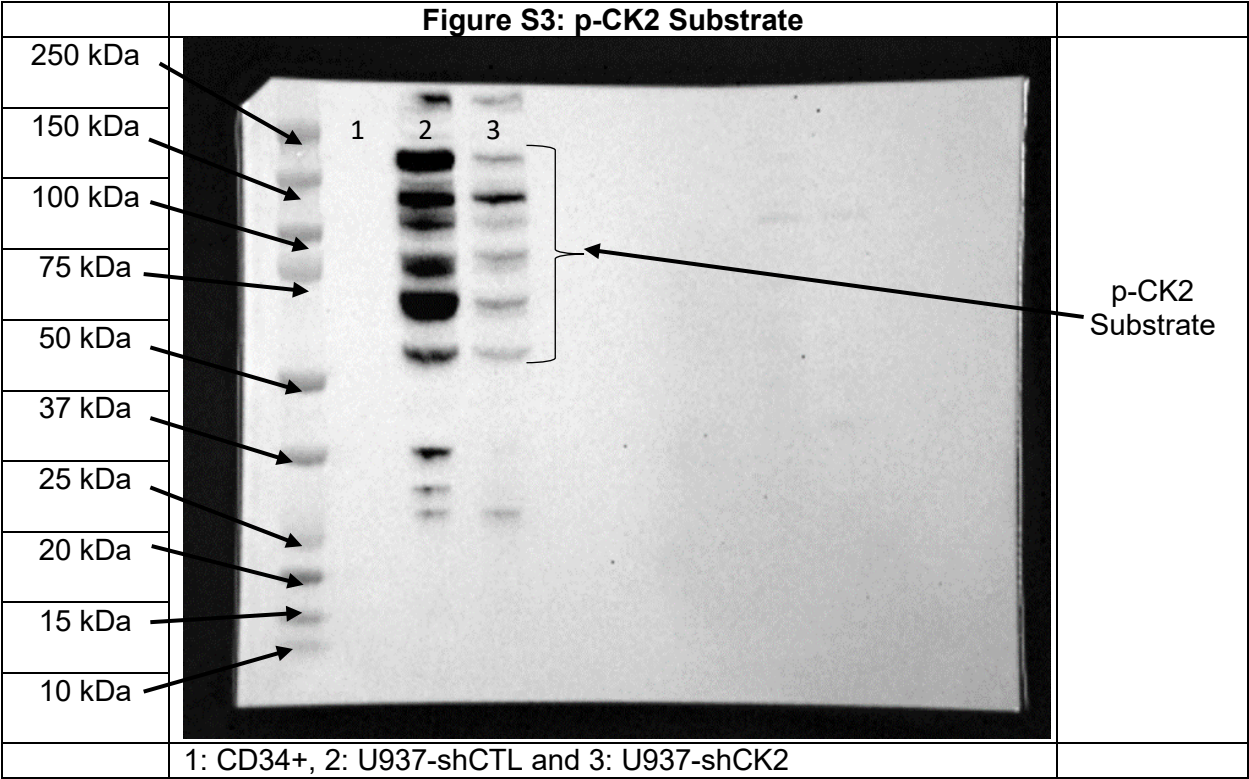

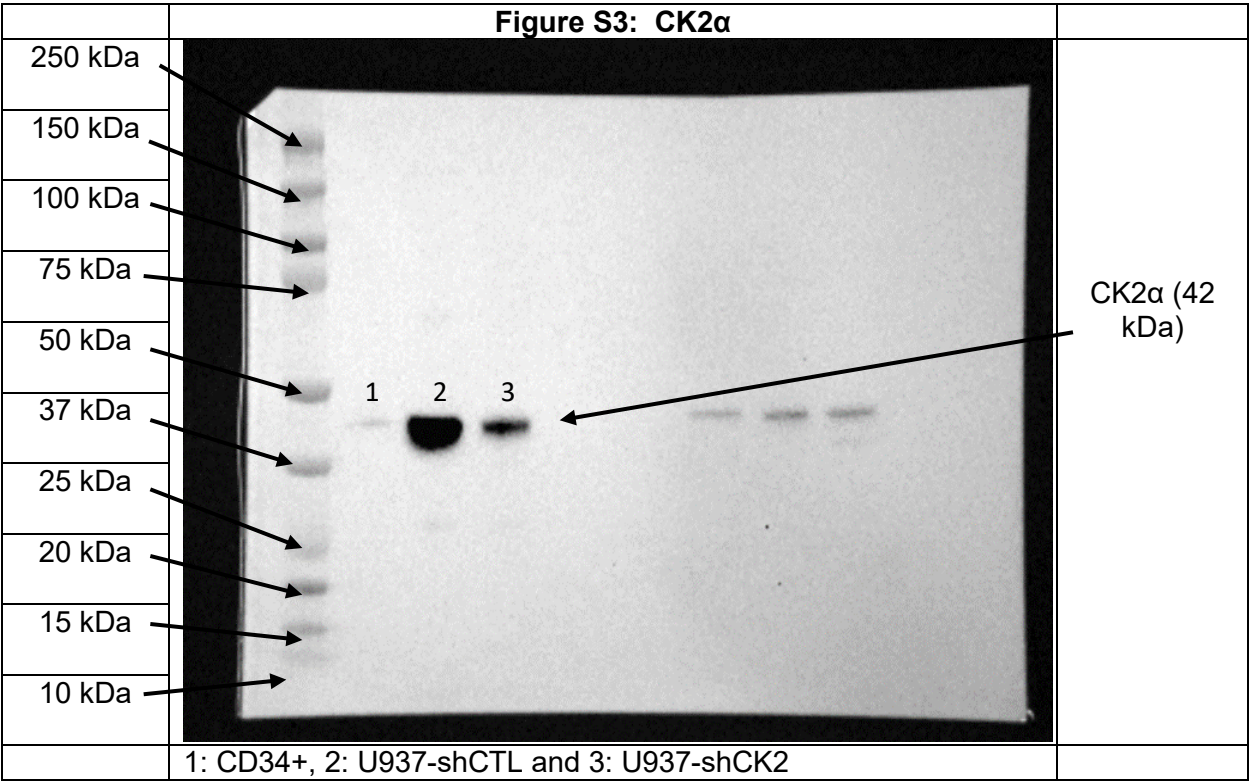

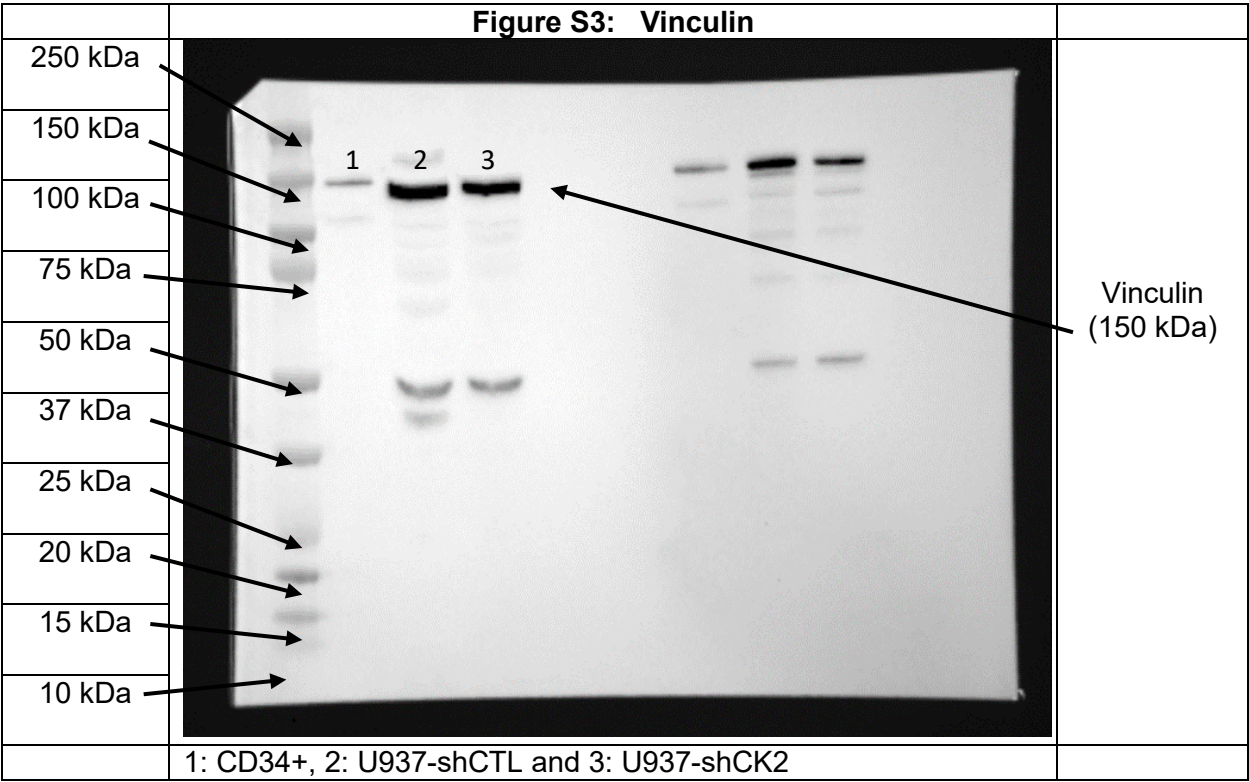

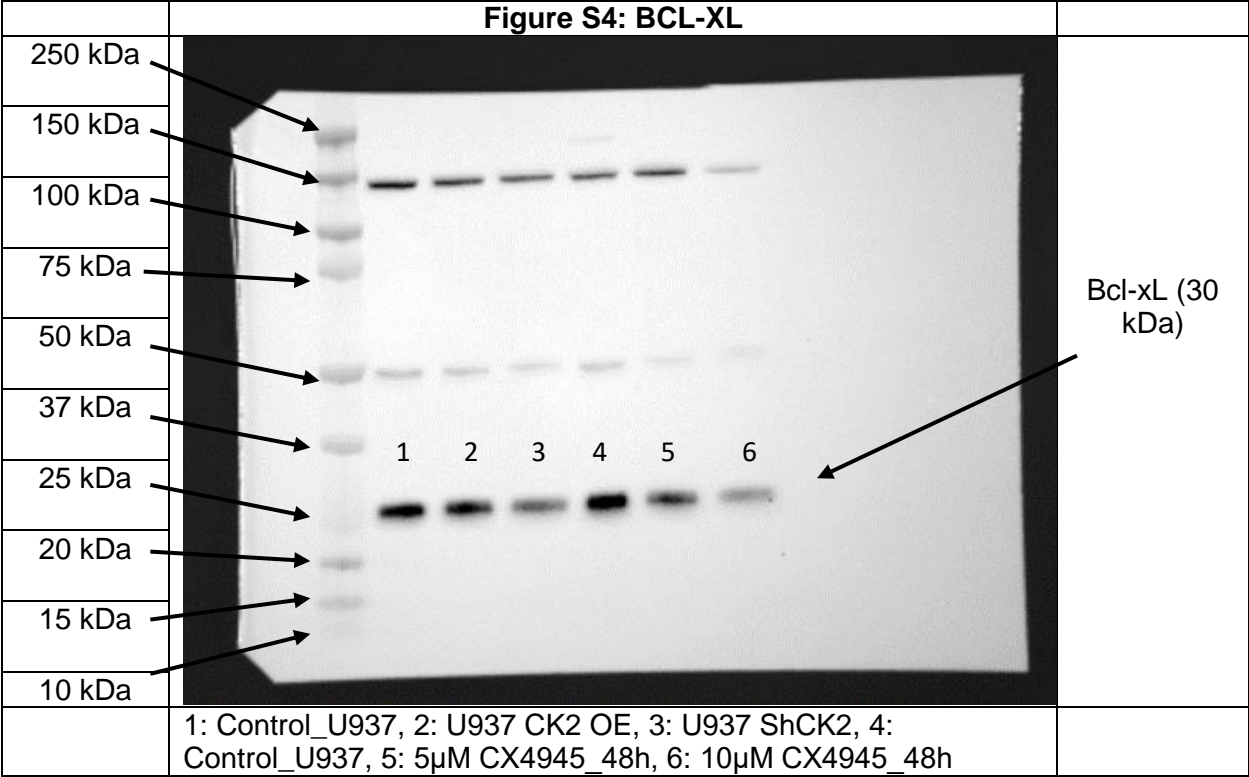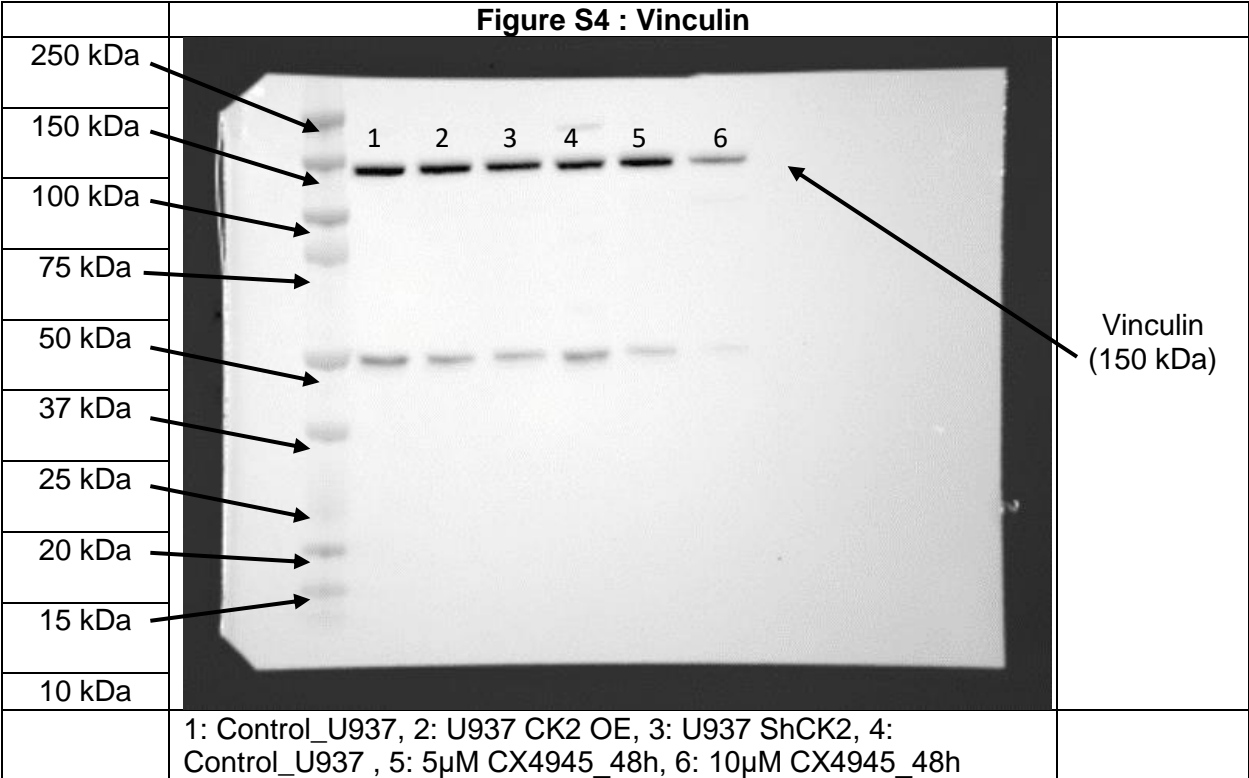

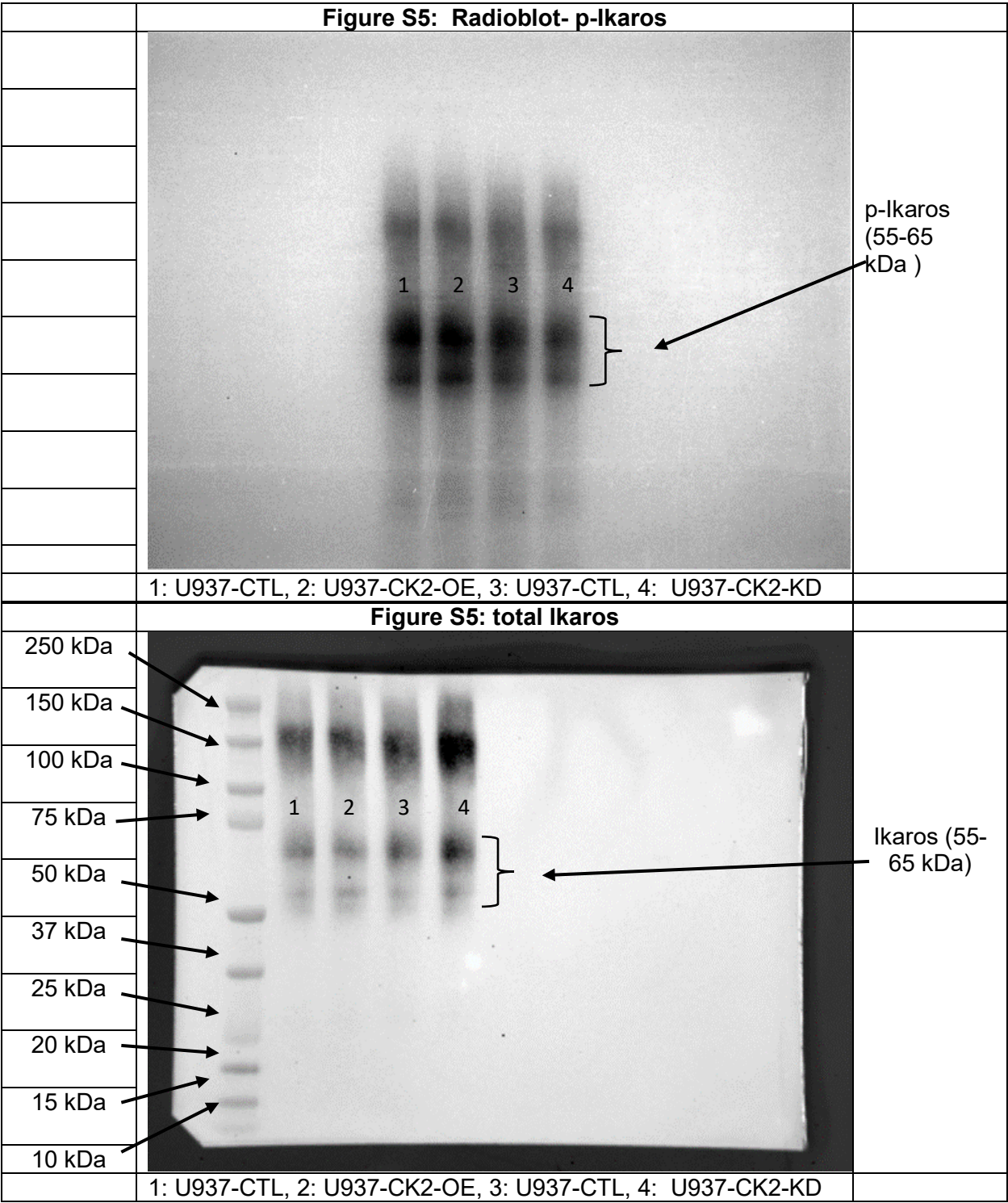

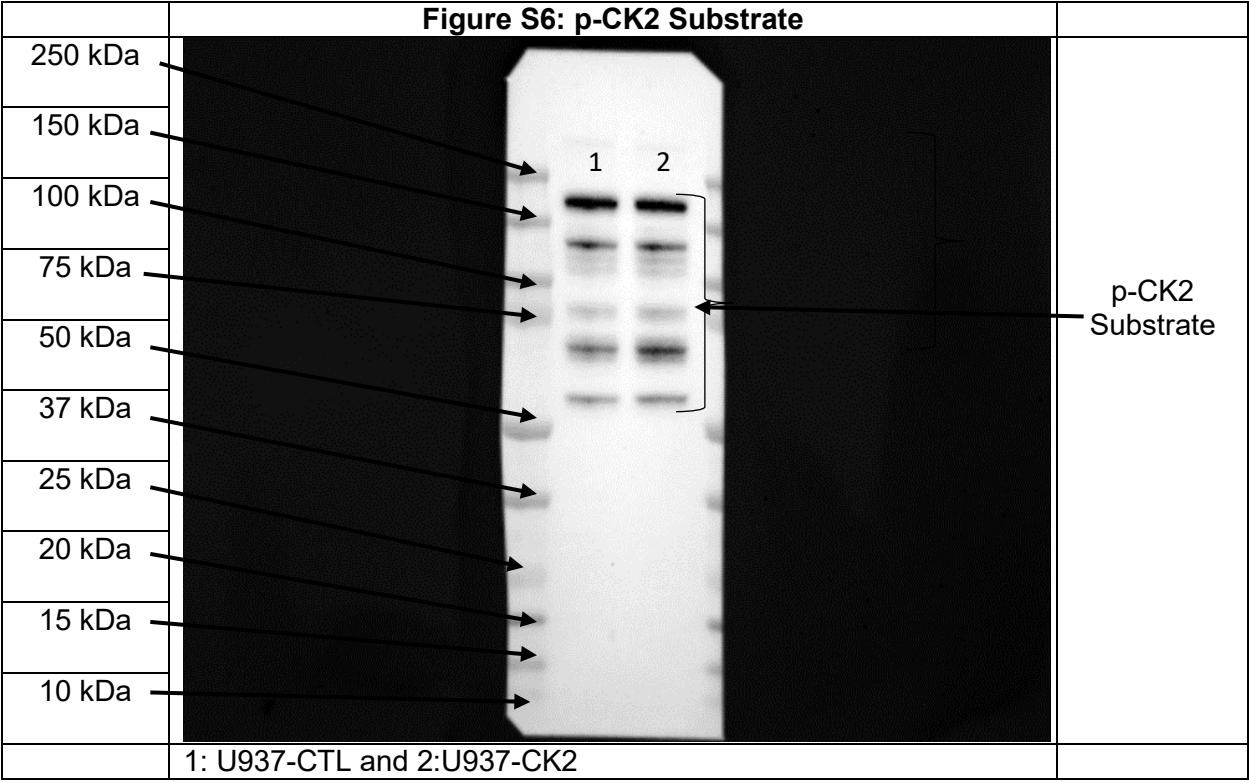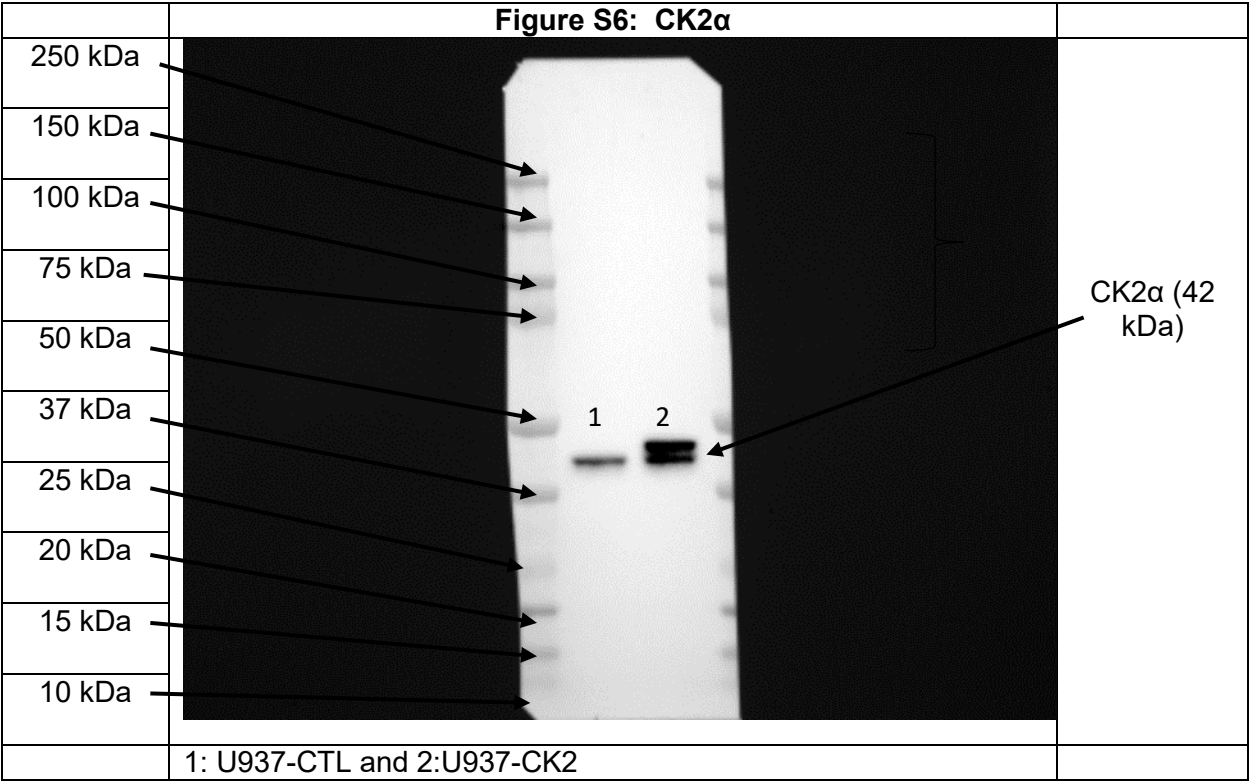

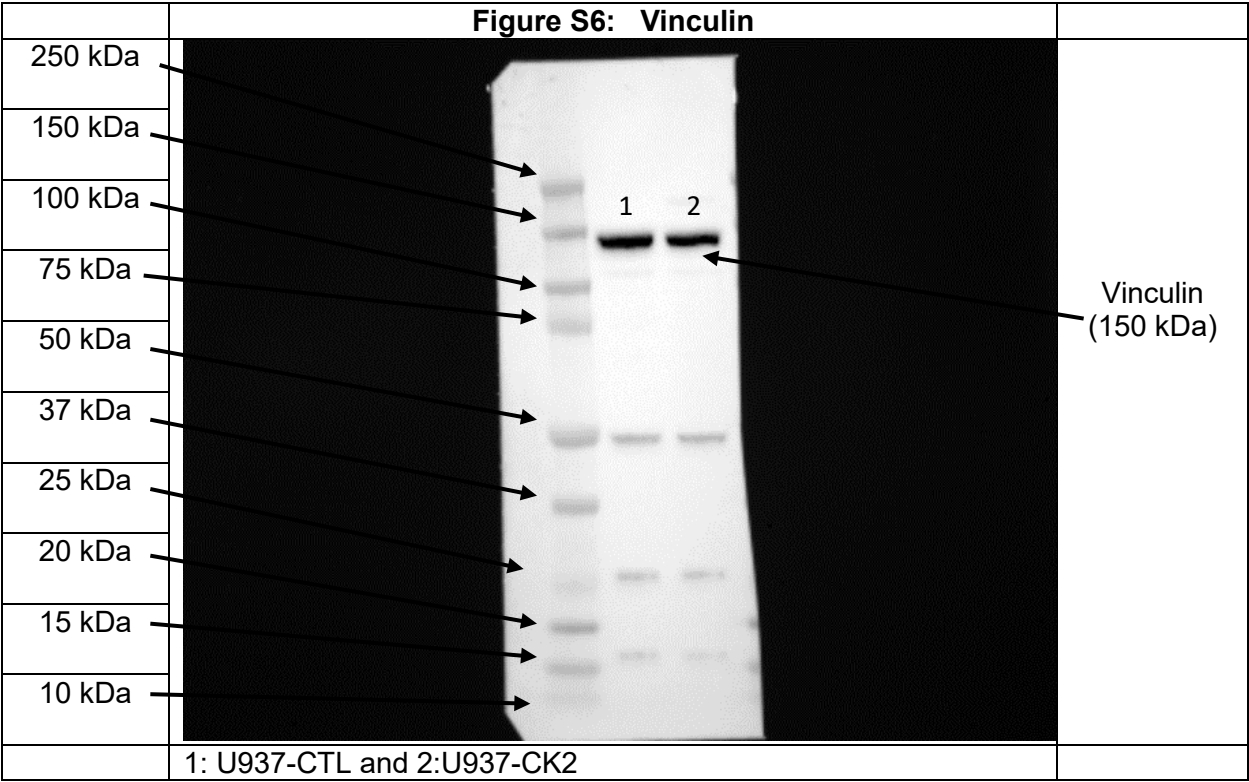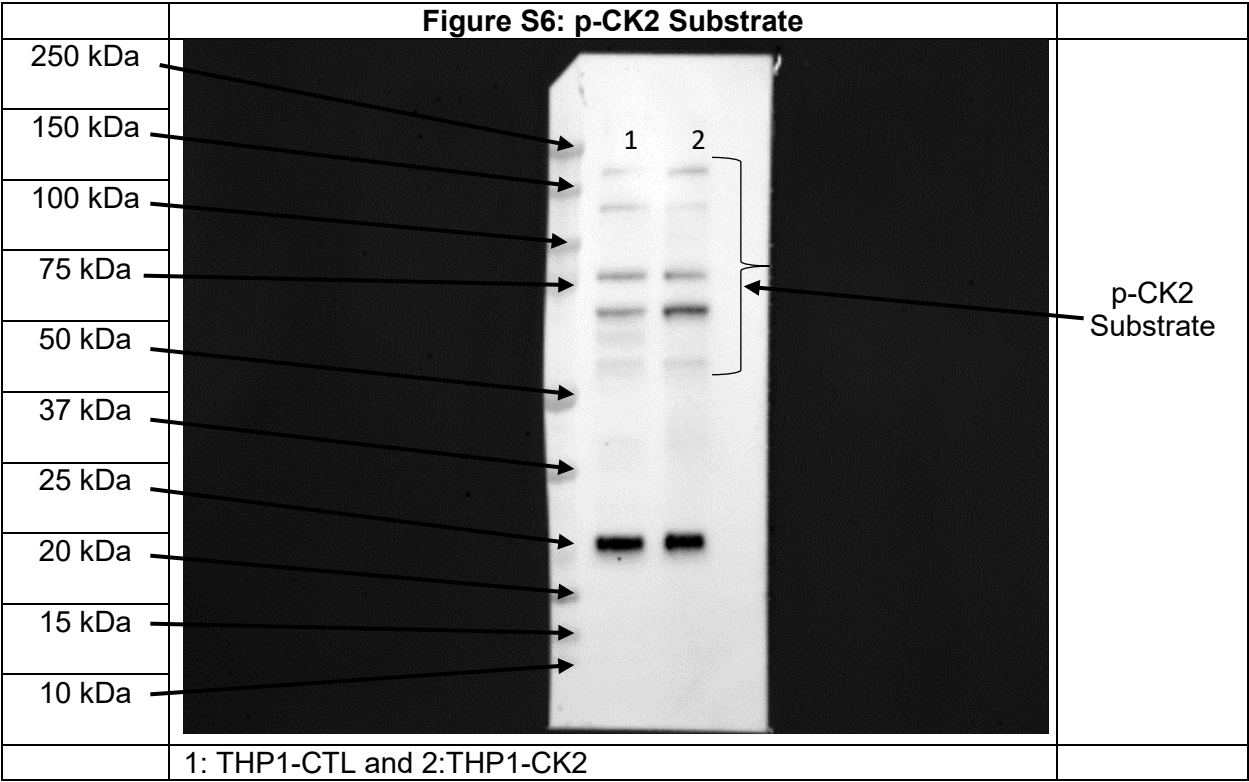

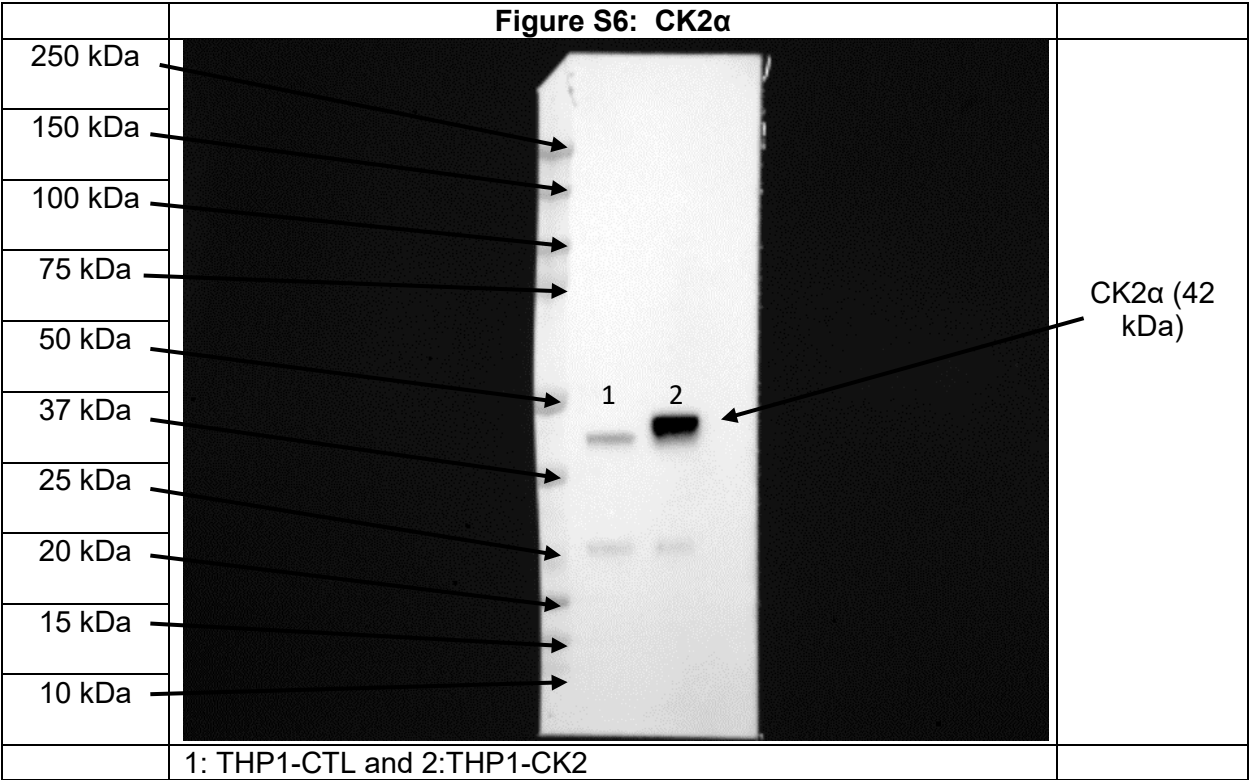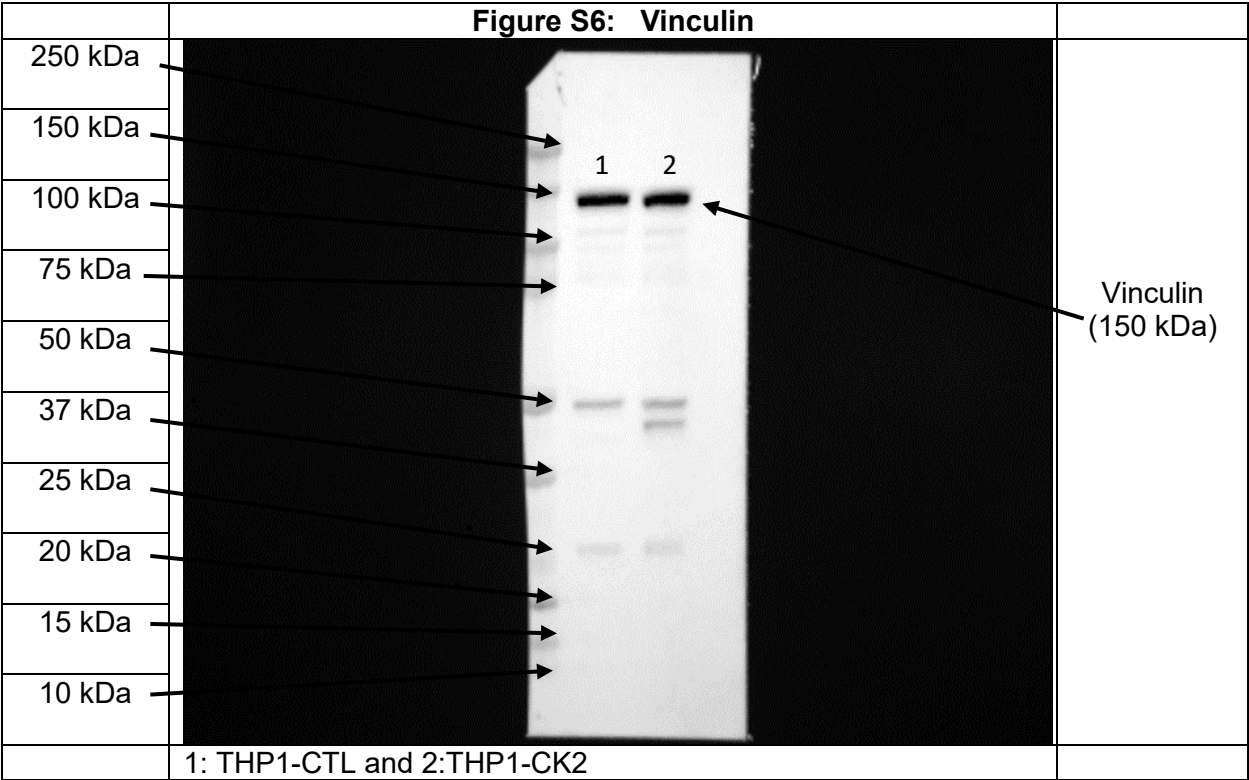

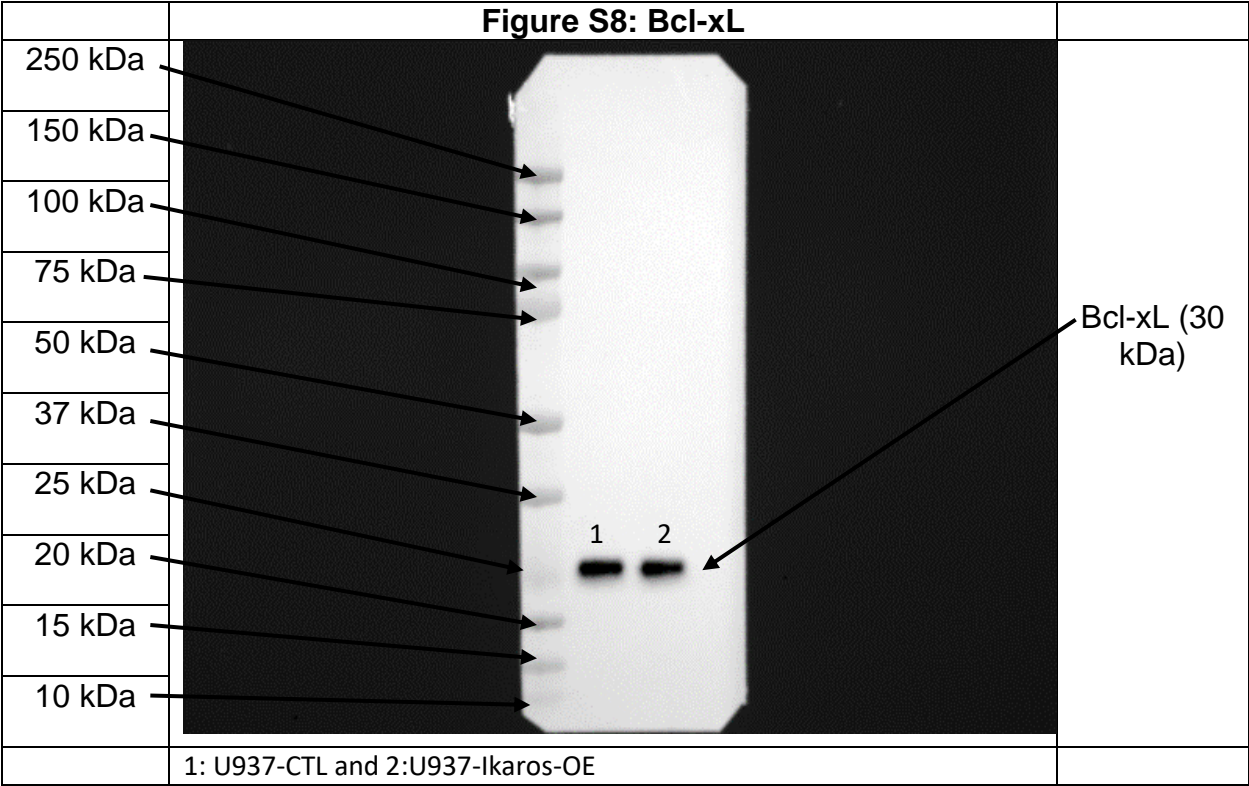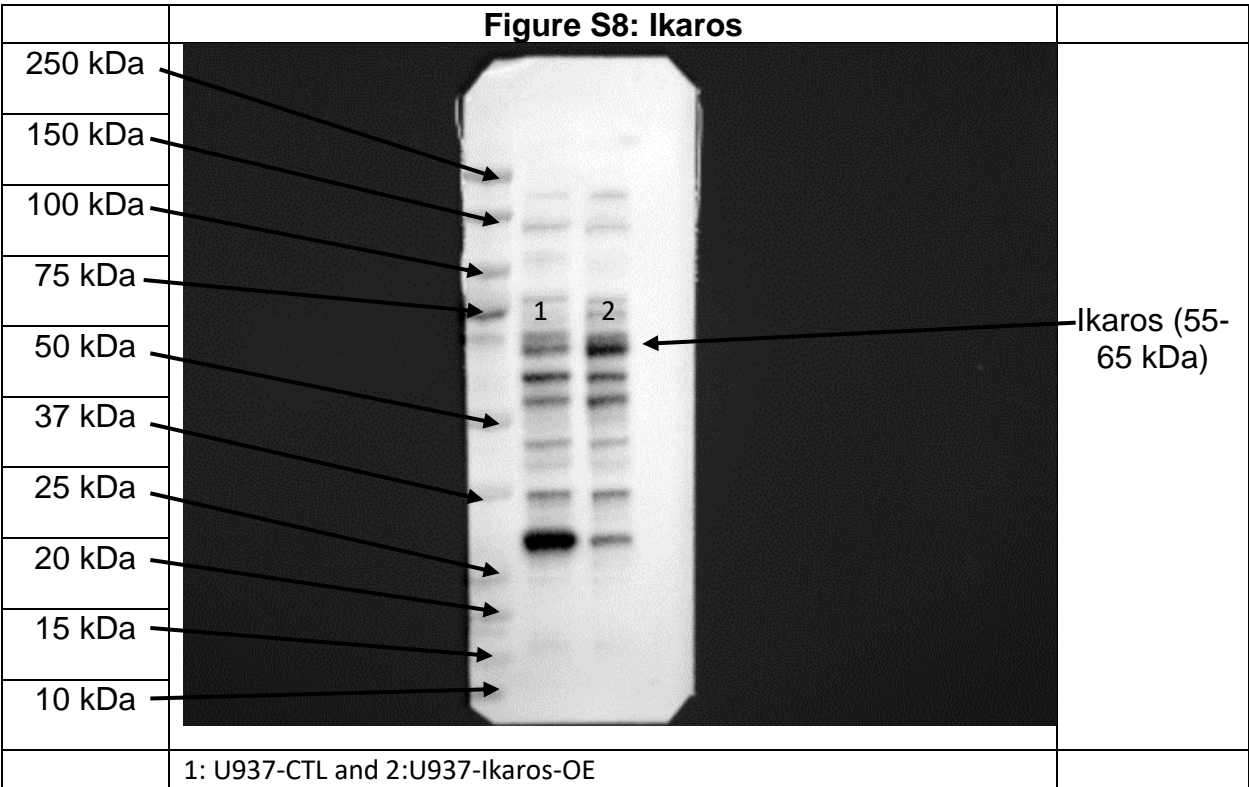

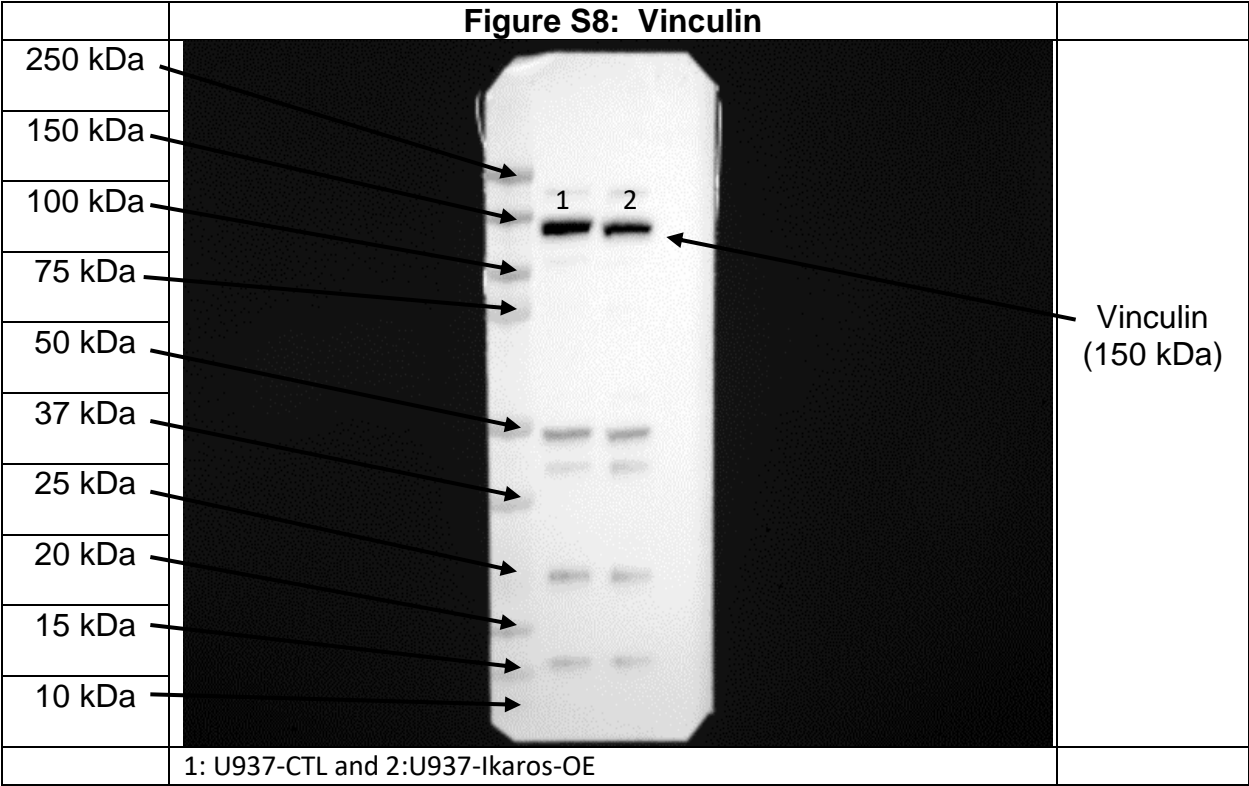

Supplement: Supplementary file 1 [file cancers-13-01127-s001.zip › 7952-supply/cancers-1097952-Western blot.pdf]
